# Telodendrimer nanotrap for selective cytokine removal from sepsis patient plasma

**DOI:** 10.3389/fimmu.2026.1633723

**Published:** 2026-03-25

**Authors:** Jennifer M. Messina, Dandan Guo, Changying Shi, Natalia Valenzuela-Faccini, Qinghe Meng, Robert N. Cooney, Juntao Luo

**Affiliations:** 1 Department of Pharmacology, State University of New York Upstate Medical University, Syracuse, NY, United States; 2 Department of Surgery, State University of New York Upstate Medical University, Syracuse, NY, United States; 3 Upstate Sepsis Interdisciplinary Research Center, State University of New York Upstate Medical University, Syracuse, NY, United States; 4 Department of Microbiology and Immunology, State University of New York Upstate Medical University, Syracuse, NY, United States; 5 Upstate Cancer Center, State University of New York Upstate Medical University, Syracuse, NY, United States

**Keywords:** charge disparity, cytokines, immune modulation, nanotrap, sepsis

## Abstract

**Introduction:**

Sepsis remains difficult to treat, resulting in persistently high mortality, creating a significant clinical need for novel therapeutic approaches that provide precise immune modulation. A highly customizable telodendrimer nanotrap (TDNT) platform was developed to selectively remove inflammatory cytokines based on cytokine charge disparity and demonstrated improved survival in experimental murine sepsis. In this study, plasma from patients with abdominal sepsis was characterized for cytokine profiles and used to optimize the engineered TDNT resins for selective attenuation by targeting cytokine charge disparity.

**Methods:**

TDNT resins were screened against a spiked mixture of patient plasma, and three lead formulations were identified with different cytokine binding profiles. The correlation between individual patient cytokine profiles and clinical diagnosis was studied. The cytokine removal efficacy of the lead candidates was subsequently validated using 20 individual sepsis patient plasma samples for comparison with a commercial macroporous hemoperfusion resin.

**Results:**

After screening patient plasma, we optimized three lead nanotrap resins to selectively absorb either positively charged or negatively charged cytokines or to target universal cytokine removal. Upon validation in individual sepsis patient plasma, the selective cytokine binding profiles of the lead selective nanotrap resins were confirmed; however, overall cytokine clearance efficacy was moderate. The pan-affinitive TDNT resin effectively removes cytokines from patient plasma with comparable efficiency to the commercial MG250^®^ resin and significantly lower nonspecific adsorption of serum proteins, indicating more promising biocompatibility. A trend of correlation was observed between total cytokine levels in patient plasma and clinical Sequential Organ Failure Assessment (SOFA) score, as well as an inverse correlation with Systemic Immune-Inflammation Index (SII) score. Finally, the pan-affinitive TDNT resin was able to remove cytokines from patient plasma regardless of patient SOFA or SII score and effectively reduced the overall cytokine burden to levels that correlate with reduced mortality risk.

**Conclusion:**

Targeting cytokine charge disparity and total cytokine burden using TDNT resins with different cytokine binding profiles is promising for effectively addressing the dysregulated immune response in sepsis and reducing mortality, which warrants further testing in a large cohort of patients.

## 1 Introduction

Sepsis is a complex, heterogeneous condition resulting from a dysregulated host response to infection, with significant morbidity and mortality (1–4). In some patients, immune mediators such as damage-associated molecular patterns (DAMPs) and pathogen-associated molecular patterns (PAMPs) trigger excessive immune activation, leading to overproduction of cytokines known as a “cytokine storm” (5, 6), which can progress to a condition called the systemic inflammatory response syndrome (SIRS) (7). SIRS results in overwhelming inflammation, potentially causing multiple organ failure and early death in some patients (8). In initial survivors, some achieve immune homeostasis and recover, while others have persistent immune dysfunction and remain at risk for later death due to chronic critical illness (9). Importantly, the current consensus recognizes that sepsis is not purely biphasic. Many patients exhibit concurrent hyperinflammation and sepsis-induced immunosuppression early in the disease course, and immune status can oscillate over time (10). This immunosuppressive state (often termed immunoparalysis) is associated with impaired pathogen clearance and an increased risk of secondary infections and late mortality (11).

Given the heterogeneity and complexity of sepsis pathogenesis (5), treatment remains extremely challenging. Current sepsis guidelines emphasize prompt fluid resuscitation and antibiotic administration; however, despite these measures, in-hospital mortality rates remain between 20% and 40%, largely due to the inability to modulate the dysregulated inflammatory response (12). Cytokines play a critical role in the early hyperinflammatory phase of sepsis and therefore represent important targets for restoring immune homeostasis. Unfortunately, single-target therapies, such as monoclonal antibodies aimed at neutralizing individual proinflammatory cytokines, have failed to show therapeutic benefit in clinical trials for sepsis (13–16). Hemofiltration has also been used to remove inflammatory mediators in severe sepsis. Unfortunately, previously evaluated hemoperfusion approaches have failed to demonstrate significant benefit in sepsis patients (17–21), primarily due to nonspecific adsorption. Moreover, indiscriminate mediator removal may deplete not only proinflammatory cytokines but also counter-regulatory, anti-inflammatory, and immune-supportive factors, potentially exacerbating immunosuppression and undermining host defense (22). There is, therefore, a significant clinical need to develop new therapeutics for customizable immune modulation in sepsis to address the complexity and heterogeneity of the disease and reduce mortality.

We have developed a novel, highly customizable telodendrimer (TD) nanotrap (NT) platform that can be precisely engineered through the introduction of various multivalent charged and hydrophobic moieties to fine-tune synergistic interactions with proteins and drugs, resulting in efficient loading and delivery (23–28). TDNT can be engineered in hydrogel resins to display size-exclusive and charge-selective properties (29), providing a more targeted immune modulation approach to reduce off-target effects. Both resin and injectable hydrogel TDNT formulations have been shown to effectively attenuate inflammation through the adsorption of cytokines and confer a significant survival benefit in mouse models of sepsis (29, 30). Both TD formulations and the nanotrap hydrogel resin have demonstrated high biocompatibility and hemocompatibility in both *in vitro* cell culture and *in vivo* applications (24, 26, 29–31). In this study, we further optimized the chemical structure of the TD nanotrap in a hydrogel resin platform for selective cytokine adsorption from septic patient plasma to promote successful clinical translation for precise immune modulation via hemoperfusion therapy.

In this context, selective adsorption may offer a safer, precision approach compared with pan-clearance by enabling phase-tailored immune modulation, for example, preferentially reducing hyperinflammatory cytokine burden while limiting depletion of cytokines important for immune competence during immunosuppressive states. Recent analyses of hemadsorption underscore the need for patient selection and timing and motivate the development of more selective strategies (32). Recently, we have discovered that most proinflammatory cytokines are negatively charged, whereas most anti-inflammatory cytokines are positively charged (29, 33), which may play an important role in immune regulation by modulating their tissue adherence and systemic elimination. Additionally, this charge disparity provides a unique opportunity to target either group of cytokines selectively and modulate the immune response more effectively than conventional nonspecific hemoperfusion therapies or antibody therapy against a specific cytokine. We engineered a library of TDNT resins at different densities with various combinations of charged and hydrophobic moieties to optimize the platform for selective cytokine removal. Through TDNT resin screening and validation using septic patient plasma, we have identified Polyethylene glycol acrylamide (PEGA) (PEGA)-COOH_4_C4_4_ as a promising selective nanotrap for positively charged cytokines and PEGA-Arg_4_C4_4_ as a promising selective nanotrap for negatively charged cytokines, enabling cytokine removal profiles based on protein charges. These charge-selective resins are promising for complementing clinical utility by matching distinct immune states of patients for precision therapy. Interestingly, we identified a PEGA-OA_4_C17_4_ resin with oxalic acid (OA) as charge moieties that could serve as a pan-affinitive nanotrap for unanimous cytokine removal (both positively and negatively charged cytokines), comparable to the commercial resin but with significantly reduced off-target adsorption of essential plasma proteins and drug molecules. Although some individual cytokines, e.g., interleukin (IL)-6 and IL-10, have been identified as biomarkers for sepsis outcome prediction (34, 35), the specific attenuation of these cytokines via antibodies has failed to decrease the mortality rate of sepsis in the clinic (13, 14, 36, 37). In this study, we take a unique angle to classify cytokine profiles and total cytokine burden in septic patients based on the charge disparity and examine their potential correlation with disease severity. Furthermore, we evaluate whether the engineered TDNT resins can attenuate the dysregulated cytokine profiles and total cytokine burdens for potentially effective sepsis treatments.

## 2 Methods

### 2.1 Nomenclatures

The nomenclature of TDNT resin follows the format (density)PEGA-(charge group)_#valency_ (hydrophobic groups)_#valency_. For example, 10%PEGA-COOH_4_C4_4_ indicates that the free amino groups on the commercial PEGA resin (0.4 mmol/g) were downscaled to 10% (with 90% blocked by acetylation) for the conjugation of a TD structure containing four carboxylic acid moieties and four butyryl groups on the periphery of the dendritic TD scaffold.

### 2.2 Surgical sepsis patient cohort blood collection

The sepsis cohort in this study consists of patients ≥ 18 years old with surgical sepsis (gastrointestinal perforation or ischemia) who required surgery and were admitted to the SICU at Upstate Medical University Hospital in Syracuse, NY, USA (IRB No. 1321635, approved 2 November 2018). Sepsis-3 definitions of sepsis/septic shock were used to calculate the Sequential Organ Failure Assessment (SOFA) score using the following variables: PaO_2_/FiO_2_ ratio, Glasgow Coma Scale (GCS) score, mean arterial pressure (MAP), administration of vasopressors with type, dose, and infusion rate, serum Cr (mg/dL) and urine output, bilirubin level (mg/dL), and platelet count (3). A SOFA score ≥ 2 was used as the cutoff for inclusion in the study. After meeting the initial screening criteria, informed consent was obtained. A total of 10 mL of blood was collected from an indwelling catheter already in place (e.g., arterial line, PICC line, or central venous catheter) and immediately placed on ice to reduce cellular stress under hypoxia condition *ex vivo*. Blood was centrifuged, and plasma was collected within 1 h after blood collection. Plasma samples were stored at − 80 °C for TDNT resin screening. Systemic Immune-Inflammation Index (SII), a biomarker used to determine inflammation and thrombosis status and developed as a predictor of adverse outcomes in several disease processes, including sepsis (38), was calculated for each patient with available clinical data at the time of blood collection using the following equation:


$$\text{SII}=\;\frac{\left(\text{platelet count})(\text{ absolute neutrophil count}\right)}{\text{absolute lymphocyte count}}$$

The patient cohort for TDNT resin validation (**Table 1**) consisted of 20 patients with surgical sepsis (nine men, 11 women), with an average age of 64.2 years, an average SOFA score of 9.4, and an SII score of 2,738.6. The mortality rate in this cohort was 60%. Given the limited cohort size and the *ex vivo* nature of the experiments, gender was not considered a variable for analysis.

**Table 1:** Surgical sepsis patient population for individual screening.

Total patients	20
Male (%)	9 (45%)
Female (%)	11 (55%)
Average age ± SD (range)	64.2 ± 12.7 (37–84)
Mortality (%)	12 (60%)
Average SOFA score ± SD (range)	9.4 ± 3.2 (3–15)
Average SII score ± SD (range)	2,738.6 ± 2,170.5 (141–7,746)

### 2.3 Preparation of THP-1 macrophage supernatant for spiked plasma

THP-1 human monocytic cells (ATCC) were cultured in RPMI medium (Cytiva Life Sciences, Marlborough, Massachusetts, USA) with 10% FBS and 1% penicillin/streptomycin in a humidified 5% CO_2_ incubator at 37 °C. Cells were plated at 5 × 10^4^ cells/well in a 96-well plate and differentiated into macrophages with 10 ng/mL phorbol 12-myristate 12-acetate (PMA) (Alfa Aesar, Ward Hill, Massachusetts, USA) for 24 h. The medium was refreshed, and cells were stimulated with 50 ng/mL lipopolysaccharide from *Escherichia coli* (Sigma-Aldrich, Burlington, Massachusetts, USA) for 24 h. Supernatant was collected, pooled, and stored at − 80 °C for future use.

### 2.4 TDNT resin cytokine removal from sepsis patient plasma

#### 2.4.1 TDNT resin screening with spiked-mixed plasma

For resin library screening, spiked-mixed plasma was used to identify lead TDNT candidates with positively charged cytokine-selective, negatively charged cytokine-selective, and pan-affinitive cytokine removal profiles. Considering the heterogeneity among individual sepsis patient profiles, spiking and mixing plasma ensured an abundance of cytokines to accurately determine the binding potential of the TDNT resins. Additionally, the large plasma volume required to screen the library of resins made it infeasible to use individual patient plasma prior to the selection of lead candidates. Spiked-mixed plasma was freshly prepared by first mixing plasma from nine sepsis patients and then doping it with LPS-stimulated THP-1 supernatant at a plasma/supernatant ratio of 3:1 TDNT. PEGA control was washed with 70% ethanol, followed by PBS three times. A total of 30 mg of wet hydrogel resin and commercial MG250 resin (Baihe Medical, Guangdong, China) were weighed for incubation with 60 µL of spiked-mixed plasma for 4 h on a rotator at room temperature. Samples were centrifuged, and plasma supernatant was collected for multiplex cytokine analysis and subsequent evaluation of cytokine clearance efficacy. Spiked-mixed plasma without resin incubation served as a reference for the cytokine profile.

#### 2.4.2 TDNT resin validation with individual sepsis patient plasma

Individual patient plasma presents a heterogeneous cytokine profile, which provides a more stringent test for evaluating lead TDNT resin efficacy across a wide range of cytokine profiles. The lead positively charged cytokine-selective, negatively charged cytokine-selective, and pan-affinitive TDNT resins were incubated with 60 µL of individual sepsis plasma from 20 patients for 4 h on a rotator at room temperature. Due to the limited volume of individual patient plasma, seven of the 20 samples were selected as a representative of the cohort for incubation with the MG250 commercial control resin for comparison with TDNT. Samples were centrifuged, and plasma supernatant was collected for multiplex cytokine analysis and subsequent evaluation of cytokine clearance efficacy, as well as total protein analysis via UV absorption. Individual sepsis patient plasma without resin incubation served as a reference for cytokine profile and total plasma protein level.

### 2.5 Multiplex cytokine analysis

MILLIPLEX^®^ Human Cytokine/Chemokine/Growth Factor Panel A (Millipore, Burlington, Massachusetts, USA, HCYTA-60K-18; Granulocyte-macrophage colony-stimulating factor (GM-CSF), IL-10, IL-13, IL-17A, IL-18, IL-1RA, IL-1a, IL-1B, IL-2, IL-3, IL-4, IL-6, IL-7, IL-8, MCP-1, Macrophage Inflammatory Proteins (MIP)-1α, Macrophage Inflammatory Proteins (MIP)-1β, tumor necrosis factor alpha [TNF-α]) was performed according to manufacturer’s protocol using the MAGPIX Luminex xMAP^®^ system (Millipore) for cytokine analysis. Luminex xPONENT^®^ software version 4.3 (Millipore) was used for data acquisition. For mixed plasma library screening, 14 cytokines—five positively charged (IL-8, IL-10, IL-17A, MCP-1, and interferon-γ [IFN-γ]) and nine negatively charged (GM-CSF, IL-1α, IL-1β, IL-6, MIP-1α, MIP-1β, TNF-α, IL-27, and IL-1RA)—were included for analysis based on the detectable cytokines present in the pooled plasma. For individual sepsis patient profiling and screening, 15 cytokines—six positively charged (IL-7, IL-8, IL-10, IL-13, IL-17A, and MCP-1) and nine negatively charged (GM-CSF, IL-1α, IL-1β, IL-1RA, IL-6, IL-18, MIP-1α, MIP-1β, and TNF-α)—were included to cover more cytokines detected in individual patient plasma. Cytokines below the level of detection of the assay were classified as 0 for the purpose of calculation.

### 2.6 Cytokine profile and resin cytokine clearance efficacy analysis

**Figure 1:**
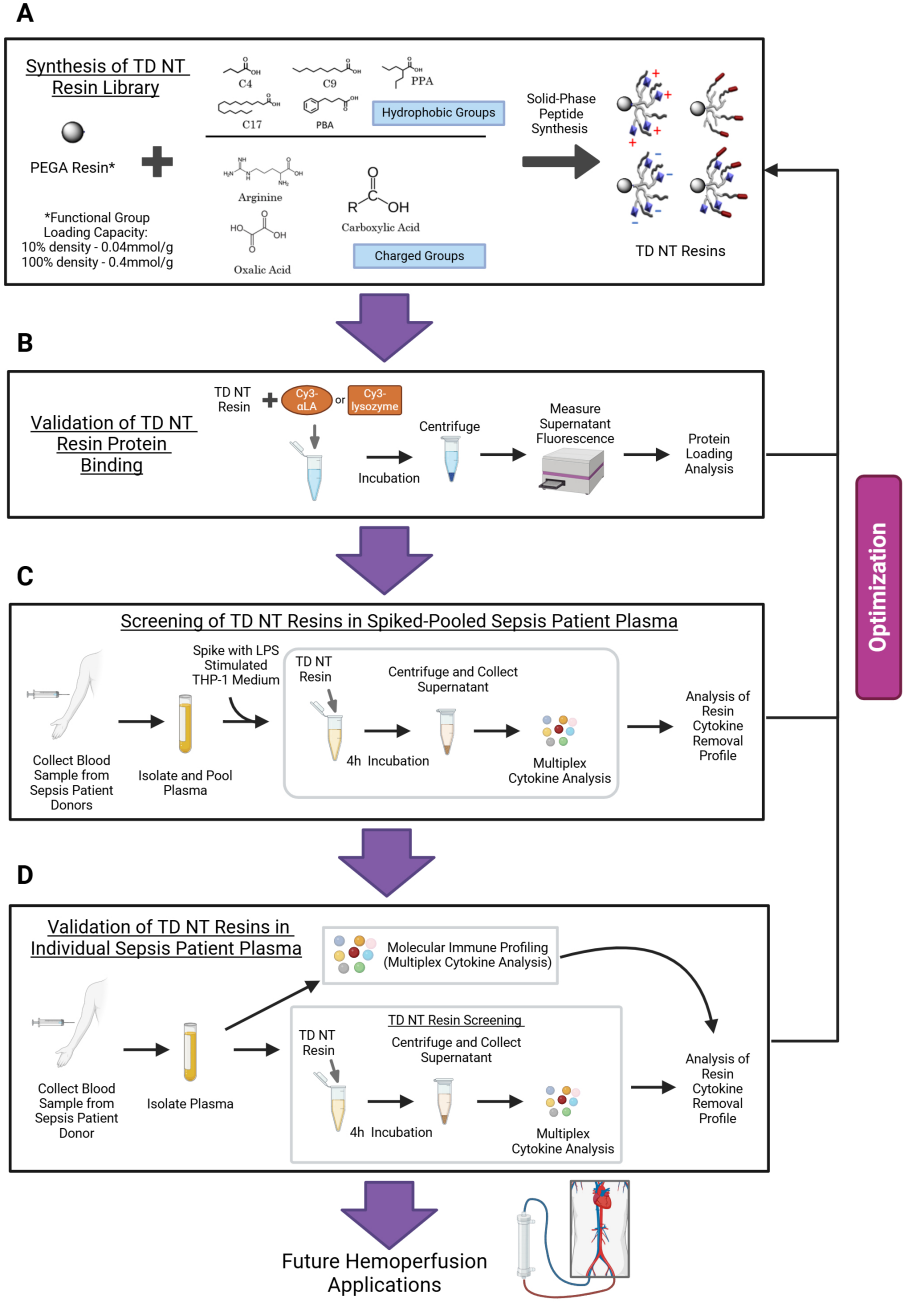
Telodendrimer (TD) nanotrap (NT) resin engineering design for selective cytokine targeting. Schematic of the engineering and validation process for the development of TDNT resins for cytokine targeting. **(A)** Generation of a library of PEGA-based TDNT resins with charged-only, hydrophobic-only, or charged and hydrophobic moieties via solid-phase peptide synthesis for charge-selective protein binding. **(B)** Validation of TDNT resin protein-binding selectivity using Cy3-αLA (negatively charged) and Cy3-lysozyme (positively charged) as model proteins. **(C)** Screening of TDNT resins in pooled sepsis patient plasma to identify leading candidates with pan-affinitive cytokine, positively charged cytokine-selective, and negatively charged cytokine-selective binding profiles. **(D)** Validation of leading TDNT candidates’ cytokine removal efficacy in plasma from 20 individual sepsis patients. The graphic was created using Biorender.com.

Cytokine profiles for mixed patient plasma and individual sepsis patient plasma were determined using a multiplex cytokine assay before and after TDNT resin incubation to evaluate individual cytokine adsorption and cytokine profile clearance by TDNT resins. Total cytokine represents the sum (by weight, **Figure 1B**) of all cytokines; positively charged cytokines represent the sum of all positively charged cytokines with an isoelectric point (*p*I) > 7.4 (**Table 2**); and negatively charged cytokines represent the sum of all negatively charged cytokines with a *p*I < 7.4 (**Table 2**), based on published predicted *p*I values in the PhosphoSitePlus database (39). The isoelectric point cutoff for classifying cytokine charge was determined based on the average human blood pH of 7.4. For individual sepsis patient plasma, additional analysis relating cytokine profile to mortality and clinical markers (e.g., SOFA score and SII) was performed. Patients were divided into high/low cytokine, SOFA score, and SII status. Cytokine levels at the 50th percentile (14,318 pg/mL for total cytokines, 4,055 pg/mL for positively charged cytokines, and 10,263 pg/mL for negatively charged cytokines), a SOFA score of 6.5 (40), and an SII of 1,767 (38) were used as cutoffs to determine high/low status.

**Table 2:** Cytokines evaluated for sepsis patient profiles and TDNT resin screening using multiplex cytokine analysis.

Cytokine	Isoelectric point (*p*I)^a^	Charge at pH 7.4
IL-18	4.54	−
IL-1β	4.7	−
MIP-1α	4.77	−
IL-1α	5.04	−
MIP-1β	5.13	−
GM-CSF	5.21	−
IL-1RA	5.83	−
TNF-α	5.89	−
IL-6	6.17	−
IL-27	6.18	−
IL-10	8.19	+
IL-13	8.69	+
IL-17A	8.82	+
IL-7	8.87	+
IL-8	9.1	+
MCP-1	9.4	+
IFN-γ	9.5	+

a Isoelectric point (*p*I) prediction of human protein from the PhosphoSitePlus Database (39).

Resin cytokine clearance efficacy (CE) for total, positively charged, and negatively charged, and individual cytokines from mixed or individual sepsis patient plasma was calculated for each resin using the following equation:


$$\%\text{ Cytokine CE}=(1-\frac{\left[{\text{Cytokine}}_{\text{resin plasma}}\right]}{\left[{\text{Cytokine}}_{\text{control plasma}}\right]})\times 100$$

Additionally, ratios of clearance efficacy of positively charged/negatively charged cytokines and negatively charged/positively charged cytokines were calculated to evaluate resin charge selectivity for cytokine removal in individual sepsis plasma samples.

### 2.7 Total plasma protein analysis

After 4 h of incubation on a rotator at room temperature with or without TDNT or commercial resin, the total protein concentration of the plasma was measured via UV absorbance at 280 nm using a NanoDrop. Percent plasma protein removal by resins for each patient sample was calculated using the following equation:


$${\%\text{Removal}}_{\text{protein}}=\frac{\left([{\text{Protein}}_{\text{control plasma}}]-[{\text{Protein}}_{\text{resin plasma}}]\right)}{\left[{\text{Protein}}_{\text{control plasma}}\right]}\times 100$$

### 2.8 Data management and statistics

De-identified clinical information, e.g., SOFA score and SII score, was collected on the day of blood collection. Patient outcomes, e.g., hospital mortality due to sepsis, were followed until discharge. All de-identified patient data were managed according to the IRB protocol. Results are reported as mean ± SD. Graphs and statistical analyses were performed using GraphPad Prism Software, version 10.2.3 (GraphPad Software). Cytokine clearance efficiency data between resins were compared using one-way ANOVA followed by Dunnett’s multiple comparisons test. Results were considered significantly different when *p* < 0.05.

## 3 Results

### 3.1 Screening of TDNT resins for effective and selective removal of cytokines in sepsis patient plasma

We engineered TDNT with diverse charge and hydrophobic combinations, as well as varying TD density on the PEGA resin, to screen TDNT for charge selectivity in protein binding (**Figure 1A**; **Supplementary Figure 1**). Two abundant proteins with a molecular weight < 30 kDa and a *p*I either higher or lower than neutral pH 7.4, chosen to mimic cytokines, were selected to screen the TDNT resin library (**Supplementary Figure 2**). Alpha-lactalbumin (αLA) from bovine milk (MW: 14.2 kDa, *p*I: 4.8, Sigma-Aldrich) served as a negatively charged model protein to mimic key proinflammatory cytokines (TNF-α, IL-1β, IL-6, etc.), while lysozyme (MW: 14 kDa, *p*I: 11.4, MP Biomedicals, Solon, Ohio, USA) served as a positively charged model protein to mimic anti-inflammatory cytokines (IL-10, IL-4, etc.). Through the adsorption screening studies on these model proteins, we confirmed that TDNT resins with charged and combined charged/hydrophobic moieties preferentially capture proteins with opposite charges (**Supplementary Figure 3**). At the same time, lower protein-capturing capacity was observed in TDNT resins containing only hydrophobic moieties, which resembles the molecular mechanisms of conventional polystyrene-based hemoperfusion devices (41). In general, the density of TD in the hydrogel resin synergizes with charge-only TDNT in capturing proteins with opposite charges. High TD density reduces protein-capturing efficiency in TDNT with both hydrophobic and charge moieties, especially for long fatty acid moieties, due to reduced swelling properties of the hydrogel resin in water. These results suggest that TDNT resins with charged and combined charged/hydrophobic moieties are more effective for charge-selective protein capture and require further optimization and validation for capturing cytokines in patient blood.

During sepsis, excessive cytokines are produced and self-perpetuated, a phenomenon known as a “cytokine storm”, which significantly contributes to immune dysregulation, organ failure, and early death in some patients (5–8). To validate the effectiveness and selectivity of TDNT resins for the removal of various cytokines in patient blood, we screened a library of TDNT formulations using sepsis patient plasma. Blood was collected, and plasma was isolated from a cohort of surgical sepsis patients at Upstate University Hospital with gastrointestinal perforation or ischemia. Given the heterogeneity of sepsis patients’ immune response, individual patients may have vastly different cytokine profiles. Plasma from nine sepsis patients was therefore mixed to ensure the presence of key cytokines involved in the innate immune response and to allow a normalized comparison between different resins. Additionally, mixed plasma was spiked (spiked-mixed plasma) with supernatant from LPS-stimulated THP-1 macrophages to ensure the presence of early-phase mediators, e.g., TNF, which may otherwise be low in later-stage sepsis patients and hinder the evaluation of their removal in TD resin screening. Using spiked-mixed plasma, we performed TD resin screening to identify the lead TDNT resins with different cytokine binding profiles (1): pan-affinitive (2), positively charged-cytokine selective, and (3) negatively charged-cytokine selective. As shown in **Figure 1A**, several key cytokines were detected at representative concentrations in the spiked-mixed septic plasma: TNF-α (4.14 ng/mL), IL-6 (7.25 ng/mL), IL-1β (0.48 ng/mL), and IL-10 (0.25 ng/mL). Notably, IFN-γ and IL-17A were only minimally detectable at 2–10 pg/mL, even in the spiked-mixed plasma of this specific cohort of severe patients with surgical sepsis. These cytokines are primarily secreted by activated T cells and NK cells in infections and autoimmune diseases (42, 43) and can be downregulated by lymphopenia in acute sepsis (44). Interestingly, very high chemokine concentrations were detected in the mixed plasma, e.g., MIP-1α (5,147 ng/mL; *p*I: 4.77) and IL-8 (104 ng/mL; *p*I: 9.1), as well as MIP-1β (6 ng/mL; *p*I: 5.13) and MCP-1 (5.5 ng/mL; *p*I: 9.4). In sepsis, these circulating chemokines are associated with organ damage and coagulopathy; however, they are not robust biomarkers for mortality in septic shock.

**Figure 2:**
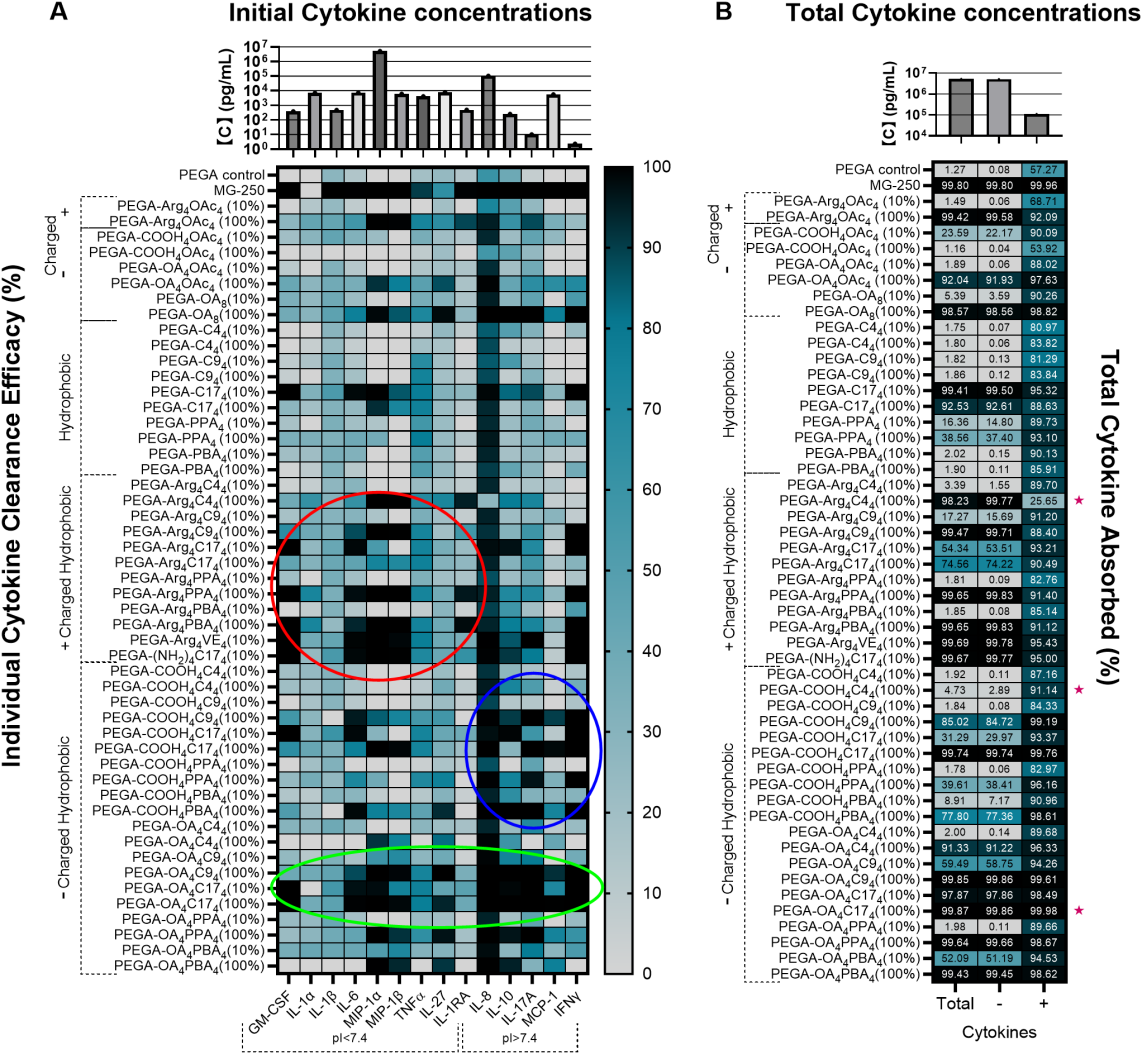
TDNT resin screening using spiked-pooled sepsis patient plasma. **(A)** Individual cytokine concentrations in the spiked-mixed plasma and the heat map of TDNT resin cytokine clearance efficacy for individual cytokines in spiked-mixed plasma from nine sepsis patients. Cytokines with a *p*I < 7.4 (negative charge) are on the left, and cytokines with a *p*I > 7.4 (positive charge) are on the right. Red circle marks the negative cytokine selective cluster; blue circle marks the positive cytokine selective cluster; the green circle marks the pan-affinitive cytokine binding cluster. **(B)** Total cytokine concentrations in the spiked-mixed plasma and the heat map of cytokine clearance efficacy analysis of total (14), negatively charged (nine), and positively charged (five) cytokines. Lead candidates selected for validation in individual sepsis plasma are denoted with. PEGA alone without TDNT serves as a negative control, and commercial MG250^®^ resin serves as a positive control.

TDNT resins were incubated with spiked-mixed sepsis patient plasma for 4 h at room temperature on a rotator. Blank PEGA control resin and a commercial MG250^®^ resin (hydrophilic-coated macroporous crosslinked polystyrene resin) were applied as negative and positive controls for comparison. After incubation, samples were centrifuged, and the supernatant was collected for multiplex cytokine analysis (**Figure 1C**). Spiked plasma without resin incubation served as an untreated reference for calculating resin cytokine clearance efficacy. The multiplex cytokine panel included nine negatively charged cytokines (*p*I < 7.4) and five positively charged cytokines (*p*I > 7.4) produced during sepsis inflammation. Resin cytokine clearance efficacy was calculated for each individual cytokine (**Figure 2A**). Similar to the model protein binding shown in **Supplementary Figure 3**, TDs with only charge or hydrophobic moieties generally exhibit poor cytokine adsorption, except for TDs with higher charge density (OA_8_ or Arg_4_ at 100%) or longer lipid chains (C17_4_ at 10%). As shown in **Figure 2A**, clusters of TD resins, indicated by different colored circles, can be identified for selective cytokine adsorption based on charge disparity, although this selectivity is not as exclusive as observed for model protein adsorption, i.e., αLA and lysozyme in **Supplementary Figure 3**. Hydrophobic interactions may play an important role in specific cytokine binding, which can compromise charge selectivity. In addition, the concentrations of individual cytokines may impact removal efficiency due to the dynamic binding between cytokines and serum proteins or TD nanotraps. However, no significant trend was observed based on cytokine concentrations. For example, the abundant and positively charged chemokine IL-8 (CXCL-8) could be effectively removed by almost all types of TD resins, whereas the more abundant negatively charged chemokine MIP-1α (CCL-3) or trace amounts of positively charged IFN-γ could only be removed by TDNT resins with a specific charge/lipid combination. Additionally, IL-1α and IL-1β, both present at moderate concentrations, were only moderately removed by all TD resins and the commercial MG250^®^ resin via nonspecific hydrophobic interactions.

In order to further explore cytokine profile selectivity, the capacity of resins to adsorb groups of positively charged cytokines (IL-8 + IL-10 + IL-17A + MCP-1 + IFN-γ) or negatively charged cytokines (GM-CSF + IL-1α + IL-1β + IL-6 + MIP-1α + MIP-1β + TNF-α + IL-27 + IL-1RA), as well as total cytokines, was analyzed in **Figure 1B**. Blank PEGA resin had very low cytokine clearance efficacy (1.27% of total cytokines), whereas the commercial resin MG250^®^ showed high cytokine clearance efficacy (99.80% of total cytokines) but lacked selectivity for differently charged cytokines (**Figure 1B**). To identify the most promising TDNT resin formulation with a pan-affinitive cytokine removal profile, total cytokine removal efficacy was compared. Several promising candidates were identified among the formulations containing a combination of charged and hydrophobic moieties that removed most cytokines from the spiked-mixed plasma. We identified 100% PEGA-OA_4_C17_4_ as the lead pan-affinitive TDNT resin, with a total cytokine removal efficacy of 99.87% (**Figure 1B**). To identify TDNT resin formulations with the most promising negatively charged cytokine-selective and positively charged cytokine-selective removal profiles, we compared the removal efficacy of all negatively charged cytokines with that of all positively charged cytokines. Thus, 100% PEGA-Arg_4_C4_4_ was the only TDNT formulation in the library that exhibited a negatively selective cytokine removal profile, removing 99.77% of negatively charged cytokines and only 25.65% of positively charged cytokines (**Figure 2B**). While several negatively charged and negatively charged/hydrophobic TDNT formulations effectively removed positively charged cytokines with minimal removal of negatively charged ones, 100% PEGA-COOH_4_C4_4_ displayed the most promising positively charged cytokine-selective removal profile, removing 91.14% of positively charged cytokines and only 2.89% of negatively charged cytokines (**Figure 1B**). The combination of charged moieties with the smaller, less bulky C4 hydrophobic group allows improved charge selectivity while maintaining efficacy through the synergistic combination of charged and hydrophobic interactions for cytokine removal. The smaller size of C4 results in fewer hydrophobic interactions than larger groups, such as C17, thereby reducing nonspecific protein adsorption and allowing a more charge-selective binding profile. Based on the screening of the TDNT resin library for cytokine removal efficacy in spiked-mixed sepsis patient plasma, we identified PEGA-OA_4_C17_4_ as the lead pan-affinitive ((pan)TDNT), PEGA-Arg_4_C4_4_ as the lead negatively charged cytokine-selective ((−)TDNT), and PEGA-COOH_4_C4_4_ as the lead positively charged cytokine-selective ((+)TDNT) formulation for further evaluation of efficacy in individual patient samples to promote effective clinical translation.

### 3.2 Cytokine profiles in sepsis patients

To effectively evaluate the therapeutic potential of these three lead TDNT resins for cytokine removal in individual sepsis patient plasma samples, it is critical to understand the cytokine profile and clinical status of the patients in the cohort. While extensive studies have evaluated the importance and prognostic value of individual cytokines such as TNF-α and IL-1β (45, 46) in the sepsis disease process, studies assessing the broader cytokine profile in sepsis patients are limited and often focus only on the combined prognostic value of a few key cytokines, e.g., IL-6 + IL-8 + MCP-1 + IL-10 (47). Here, we take a unique approach to examine a broader cytokine profile in plasma to categorize a cohort of 20 surgical sepsis patients (**Table 1**) based on the total cytokine level, positively charged cytokine profiles, and negatively charged cytokine profiles for subsequent validation of TDNT resin efficacy for cytokine removal (**Figure 1D**).

**Figure 3:**
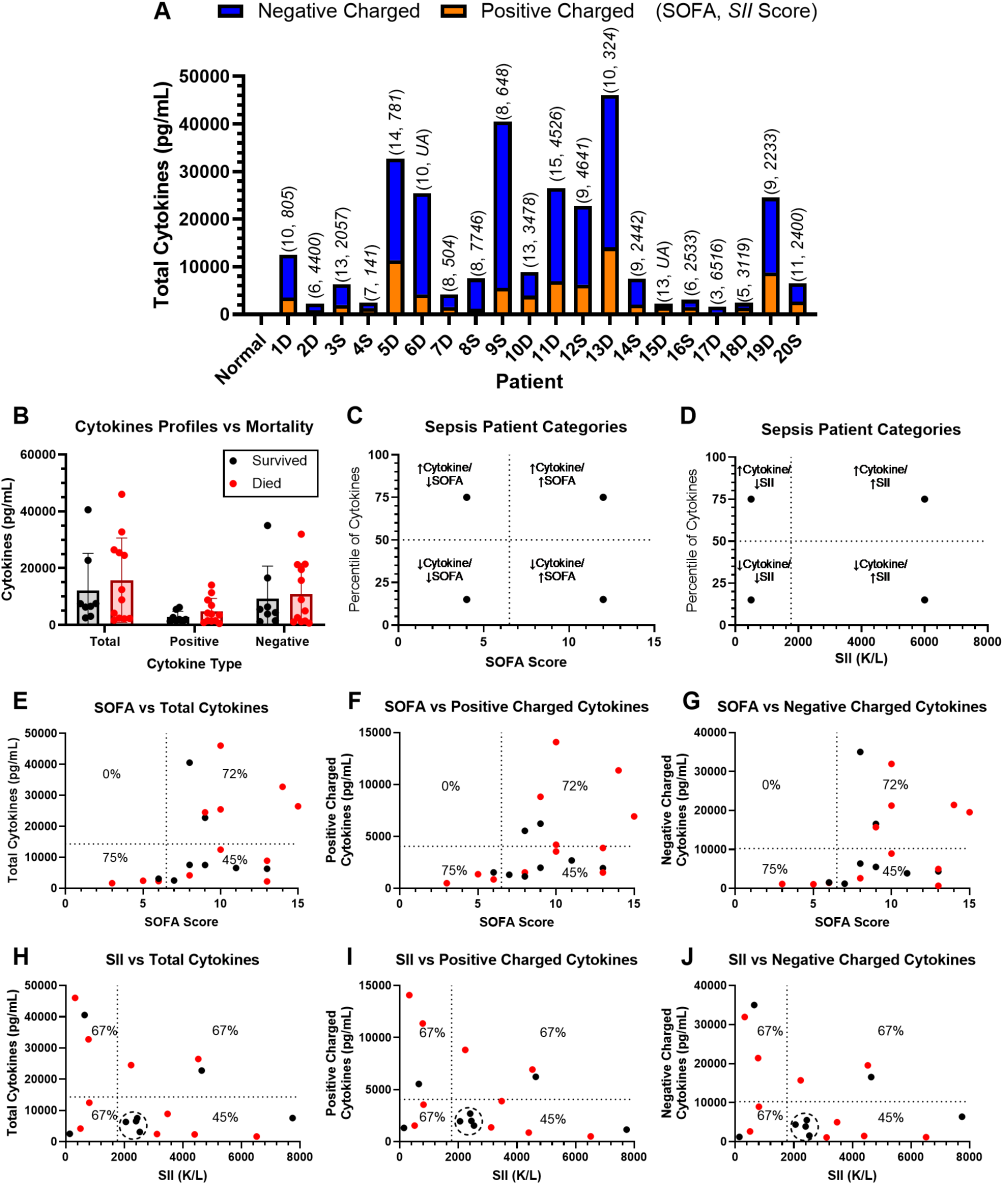
Relationship between sepsis patient cytokine profile and SOFA/SII scores. **(A)** Cytokine profiles (15 total cytokines, nine negatively charged + six positively charged) in 20 individual sepsis patients (D, dead; S, survivor) plasma samples and one healthy control were evaluated using multiplex cytokine analysis. **(B)** Comparison of total, positively charged, and negatively charged cytokine levels between survivors and nonsurvivors. Means with error bars representing SD are shown on the graph. Statistical significance was determined by a *t*-test, with significant *p*-values of < 0.05 depicted on the graphs. **(C, D)** Sepsis patients were stratified based on a combination of **(C)** SOFA score, low < 6.5 and high > 6.5, or **(D)** SII, low < 1,767 and high > 1,767, vs. cytokine profile (low < 50th percentile by weight and high > 50th percentile by weight). Correlation between patient SOFA score **(E–G)** or SII **(H–J)** and **(E, H)** total, **(F, I)** positively charged, or **(G, J)** negatively charged cytokine levels. Each symbol corresponds to an individual patient, and mortality status is indicated by black (survived) or red (nonsurvivors). Survival rates were calculated for different domains of patient classification in **(E–G)**.

Using multiplex cytokine analysis, plasma levels of 15 cytokines (nine negatively charged [GM-CSF + IL-1α + IL-1β + IL-1RA + IL-6 + IL-18 + MIP-1α + MIP-1β + TNF-α] and six positively charged [IL-7 + IL-8 + IL-10 + IL-13 + IL-17A + MCP-1]) were measured in each of the 20 sepsis patients and one healthy control plasma sample. Despite the cohort having a common septic source (gastrointestinal perforation or ischemia), significant variability was observed among the 20 patients’ cytokine profiles. Total measured cytokines ranged from 1,656 to 46,049 pg/mL, positively charged cytokines ranged from 502 to 14,084 pg/mL, and negatively charged cytokines ranged from 688 to 35,004 pg/mL (**Figure 3A**). When comparing cytokine levels in survivor vs. nonsurvivor patients, no significant differences were observed in total cytokine levels or in subgroups of cytokines based on charge disparity (**Figure 3B**). In septic nonsurvivors, both total cytokines and proinflammatory cytokines appear to deviate to either extremely high or low concentrations, indicating severe immune dysregulation, whereas cytokines in sepsis survivors are mostly clustered within mid-range levels. In addition, positively charged anti-inflammatory cytokines are higher in sepsis nonsurvivors (**Figure 3B**). Although cytokine profiles need to be confirmed in additional large cohort studies, this trend aligns with the clinical observation that both hyperinflammation and immunosuppression contribute to mortality in sepsis through either multiple organ failure or secondary infections, respectively (48–51). Immune phenotyping in sepsis is a complex clinical challenge (52–55), and systematic studies correlating patient cytokine profiles with clinical phenotypes and sepsis outcomes remain lacking. Current methods of cytokine analysis are slower than the rapid clinical progression of sepsis. In addition, specific cytokines, e.g., IL-6, may only serve as biomarkers for sepsis development, as the attenuation of specific cytokines lack clinically efficacy in reducing sepsis mortality in many large clinical trials (13, 14, 48). Therefore, the characterization of cytokine profiles in sepsis patients and the potential attenuation of groups of cytokines via engineered TDNT resins, by targeting cytokine charge disparity, may provide a novel strategy to stratify patients and enable effective and precise immune modulation therapies for sepsis patients.

To precisely diagnose the status of sepsis, both ongoing humoral immunity (secreted macromolecules in body fluids) and current cellular immunity (immune cell status) need to be evaluated in combination with organ damage (results of hyperinflammation) for improved patient management. These three parameters are interwound in determining patient outcomes in sepsis, which is further complicated by the presence of presepsis comorbidities. The SOFA score is widely used to quantify organ damage in critically ill patients and to identify those at risk. It evaluates organ dysfunction across six systems: respiratory, cardiovascular, neurological, hepatic, renal, and coagulation (40, 56, 57). Recently, the SII, which uses platelet counts (*P*), lymphocyte counts (*L*), and neutrophil counts (*N*) as a biomarker (SII = *P* × *N*/*L*) to determine inflammation and thrombosis status, has been developed as a predictor of adverse outcomes in several disease processes, including sepsis (38). Both increased SOFA score and increased SII are strongly correlated with a higher risk of mortality in sepsis patients (38, 57). Given that the SOFA score and SII are predictive of mortality in sepsis, we sought to determine whether these clinical markers have a potential relationship with patient cytokine profiles, which could provide insight into how TDNT resin therapy might be implemented in the future.

In order to study the cytokine profiles in combination with clinical diagnosis, we applied thresholds for SOFA and SII identified in large retrospective cohort sepsis studies to divide the small cohort of surgical sepsis patients into high- and low-risk groups: a SOFA score of 6.5 (40) and an SII of 1,767 × 10^9^/L (38). Patients were further divided into high- or low-cytokine groups using the 50% cytokine level in the cohort. Consequently, patients were classified into four phenotypes based on cytokine level and SOFA score: low cytokine/low SOFA, low cytokine/high SOFA, high cytokine/low SOFA, and high cytokine/high SOFA (**Figures 3C, E**). Similarly, patients were divided into low cytokine/low SII, low cytokine/high SII, high cytokine/low SII, and high cytokine/high SII to study SII–cytokine correlations (**Figures 3D, F**). Furthermore, patients were divided into subgroups based on the profiles of positively or negatively charged cytokines (**Figures 3F, G, I, J**). The mortality rate was also calculated for each sepsis phenotype in the patient cohort (**Figures 3E–J**). We observed a trend of increasing SOFA score correlating with higher total (**Figure 3E**), positively charged (**Figure 3F**), and negatively charged cytokines (**Figure 3G**). No patient in the cohort fell into the region with low SOFA/high cytokine, highlighting the association of cytokine storm with organ damage. The high SOFA/low cytokine group had the lowest mortality rate of 45% compared with both high SOFA/high cytokine and low SOFA/low cytokine, 72% and 75%, respectively (**Figures 3E–G**). These observations, however, need to be validated in a large cohort of patient populations with diverse pathogenesis and comorbidities to confirm the trend.

The reference ranges of SII in healthy controls have been reported between 142 and 804 × 10^9^/L in a large retrospective cohort study of a healthy adult population (58) and can increase during infection and inflammation. Both reduced and elevated SII indicate dysregulated immune responses, with either neutropenia/thrombocytopenia or myelosuppression/lymphopenia, which are linked to poor clinical outcomes. A retrospective cohort study of ICU patients revealed a J-shaped relationship between SII and 28-day mortality risk. An SII of 774.46 × 10^9^/L was correlated with the lowest mortality risk (59). Patients with SII greater than 1,767 × 10^9^/L were at higher risk of sepsis mortality (38), which was thus applied to subdivide the surgical sepsis patient cohort into all four SII/cytokine phenotypes. Here, we observed a trend of decreasing cytokine levels with increasing SII. In contrast, both high- and low-cytokine profiles were observed in sepsis patients with low SII, indicating greater disease heterogeneity. Patients with either high- or low-cytokine profiles and low SII had a high mortality rate of 67%. Interestingly, only the high SII/low-cytokine group exhibited a lower mortality rate of 45% (**Figures 3H–J**), with a noticeable cluster of five survivors with low cytokine and moderate SII between 2000 and 2500. Due to the small sample size, the use of a single timepoint for analysis, and high overall mortality (60%, **Table 1**), our analysis lacks statistical power to draw definitive conclusions regarding mortality trends in different SOFA/cytokine and SII/cytokine phenotypes. Despite the lack of statistical power, it is noteworthy that patients with low cytokine levels combined with both high SOFA and SII exhibited the lowest mortality rates. This observation suggests a potential survival benefit from removing these cytokines to reduce their levels in these severe patients with medium/high SII and high SOFA scores. Future analysis of a large cohort of sepsis patients is warranted to confirm the validity of trends and the potential for using cytokine profiles combined with clinical markers of SII and SOFA scores to guide personalized medicine therapies.

### 3.3 Evaluation of TDNT resin efficacy for cytokine removal in sepsis patients

Given the heterogeneous nature of the sepsis disease process and the high variability of cytokine profiles even among patients with similar septic sources (**Figure 3**), it is necessary to validate the efficacy of the lead positive-cytokine-selective (+)TDNT, negative-cytokine-selective (−)TNDT, and (pan)TDNT identified using spiked-mixed sepsis patient plasma with individual patient samples for comparison with commercial resin. Lead TDNT resins were incubated with individual plasma samples from 20 sepsis patients for 4 h at room temperature on a rotator to mimic the clinical duration of hemoperfusion therapy. Plasma from seven of those patients was incubated with the commercial MG250^®^ resin as a positive control. After incubation, samples were centrifuged, and the supernatant was collected for multiplex cytokine analysis (**Figure 1D**). Sepsis plasma without resin incubation served as a baseline reference for calculating cytokine clearance from each patient’s plasma. The multiplex cytokine panel included 15 cytokines in total: nine negatively charged cytokines (*p*I < 7.4) and six positively charged cytokines (*p*I > 7.4), as described for cytokine profiling in **Figure 3**.

**Figure 4:**
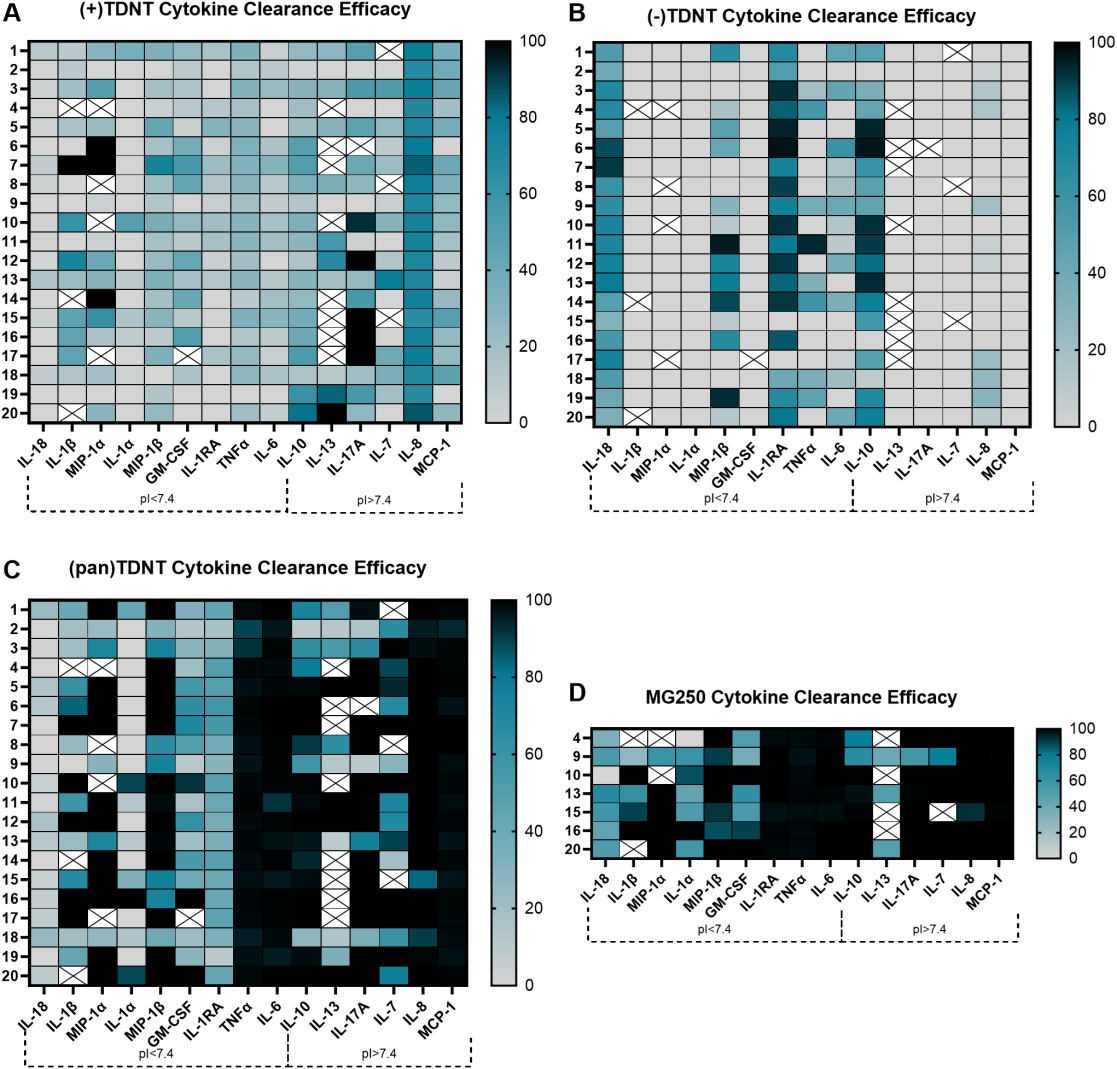
TDNT resin cytokine clearance efficacy in sepsis patient plasma. Heat map of **(A)** (+)TDNT, **(B)** (−)TDNT, and **(C)** (pan)TDNT resin cytokine clearance efficacy evaluation of individual cytokines in 20 sepsis patient plasma samples. Cytokines with a *p*I < 7.4 (negative charge) are on the left, and cytokines with a *p*I > 7.4 (positive charge) are on the right. **(D)** Commercial resin MG250^®^ was evaluated for cytokine clearance efficacy in seven patient plasma samples as a positive control for comparison. A white box with an X indicates an undetectable level of a cytokine in that sample.

**Figure 5:**
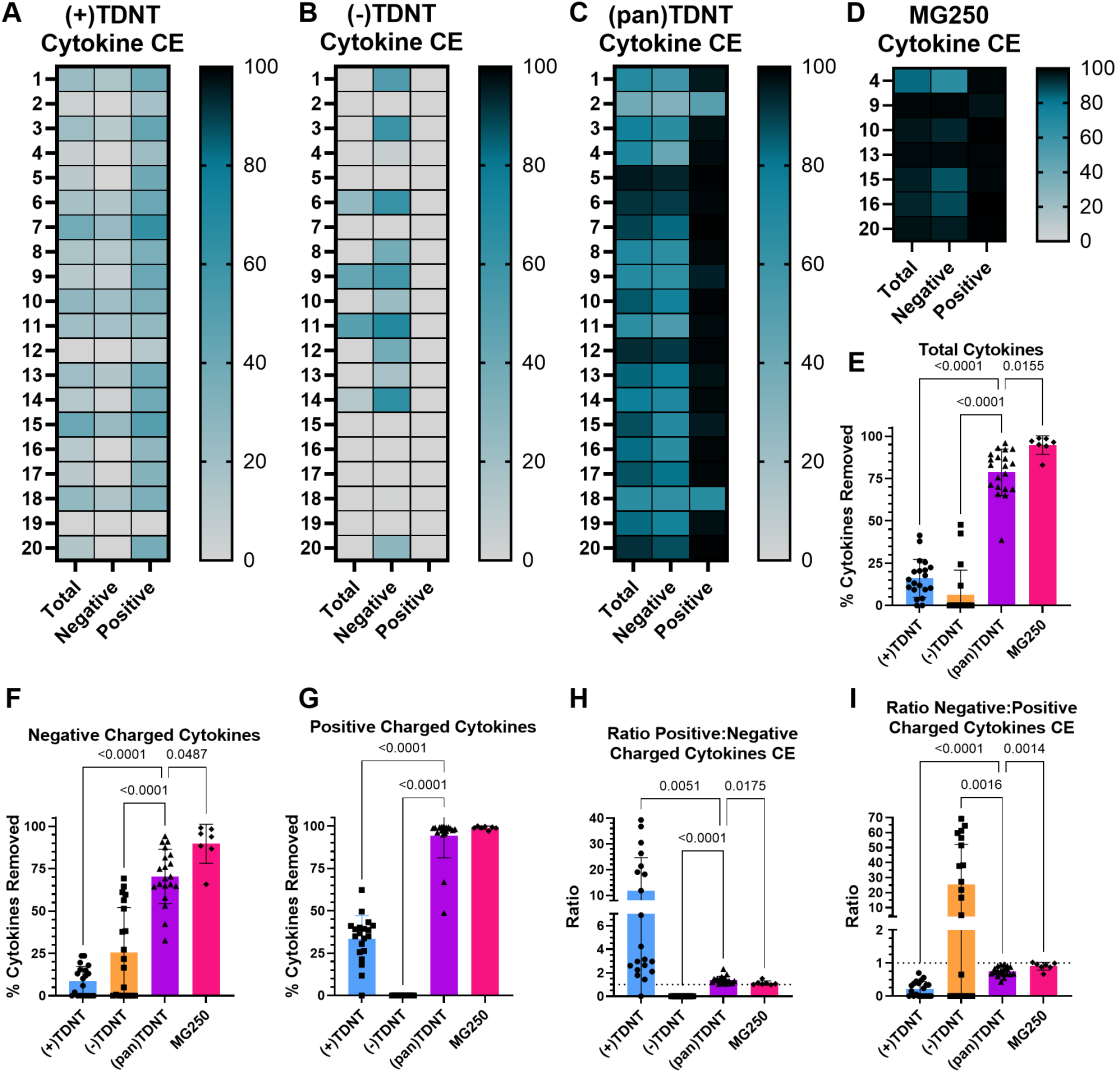
TDNT resin selectivity for cytokine removal from sepsis patient plasma. Heat map of total, positively charged, and negatively charged cytokine clearance efficacy in 20 sepsis patient plasma samples by **(A)** (+)TDNT, **(B)** (−)TDNT, and **(C)** (pan)TDNT resin. Cytokine clearance efficacy was also evaluated in seven patient plasma samples using **(D)** commercial MG250 as a positive control for comparison. Summary of **(E)** total cytokine, **(F)** negatively charged cytokine, and **(G)** positively charged cytokine removal from sepsis patient plasma by TDNT resins and the MG250 control. Ratio of **(H)** positive to negatively charged and **(I)** negative to positively charged cytokine clearance efficacy for comparison of charge selectivity of cytokine removal by resins. **(E–I)** Each symbol corresponds to an individual patient sample. Mean with error bars representing SD is shown on the graphs. Statistical significance was determined by one-way ANOVA with Dunnett’s multiple comparisons test. Significant *p*-values of < 0.05 are depicted on the graphs.

The positive-cytokine-selective (+)TDNT removed cytokines with a positive charge (*p*I > 7.4) more effectively than those with a negative charge (*p*I < 7.4) (**Figures 4A**, **5A, F, G**). When examining individual cytokines, (+)TDNT most effectively removed IL-8, IL-13, and IL-17A (**Figure 4A**; **Supplementary Figure 4**), while showing low efficacy for removal of IL-1α, IL-1RA, IL-6, and IL-18 (**Figure 4A**; **Supplementary Figure 5**). (+)TDNT had moderate cytokine removal efficacy for all other cytokines evaluated. Conversely, the negative-cytokine-selective (−)TDNT more effectively removed cytokines with a negative charge compared to those with a positive charge (**Figure 4B**; **Supplementary Figures 5B, F, G**). When examining individual cytokines, (−)TDNT most effectively removed IL-1RA, IL-18, and MIP-1β (**Figure 4B**; **Supplementary Figure 5**). Interestingly, (−)TDNT was the only formulation that could effectively remove IL-18 from sepsis patient plasma, even compared to the commercial MG250^®^ (**Figure 4**; **Supplementary Figure 5**). It also effectively removes IL-10 (positively charged) compared to (+)TDNT (**Figures 4A, B**; **Supplementary Figure 4**). (−)TDNT had moderate efficacy for the removal of TNF-α and IL-6 (**Figure 4B**; **Supplementary Figure 5**) and low efficacy for all other cytokines evaluated. Overall, both (−)TDNT and (+)TDNT showed significantly lower cytokine removal capacity than (pan)TDNT from sepsis patient plasma, which was comparable to the commercial MG250^®^ (**Figures 4C, D**, **5C–E**). The oxalic acid in (pan)TDNT can interact directly with positive amino residues in cytokines, as well as with negative amino acids via bivalent cation bridges, e.g., Ca^2+^ and Mg^2+^. In addition, the long C17 hydrophobic groups in (pan)TDNT contribute to more efficient global protein interactions. Interestingly, (pan)TDNT had poor removal efficacy for IL-1 family cytokines, e.g., IL-1α, IL-1RA, and IL-18, compared to the commercial MG250^®^ (**Figures 4C, D**; **Supplementary Figure 5**). MG250^®^ completely scavenged IL-1RA but was much less effective at removing IL-18 and inconsistent for IL-1α and IL-1β across different patients. This raises concerns for the clinical application of MG250^®^, since IL-1RA is an antagonistic ligand of the interleukin-1 receptor and inhibits multiple IL-1 family cytokines, including IL-1α, IL-1β, and IL-18 (60). Thus, the complete adsorption of IL-1RA by MG250^®^ may result in increased inflammation due to the remaining unchecked IL-1 cytokines.

(Pan)TDNT removes significantly more total cytokines than both (+)TDNT and (−)TDNT (**Figures 5E–G**). (+)TDNT, however, displayed significantly higher selectivity for the removal of total positively charged cytokines (**Figure 5H**), and (−)TDNT displayed a significantly higher selectivity for the removal of negatively charged cytokines (**Figure 5I**) compared to other TDNT formulations and the commercial MG250^®^, a trend that was consistent across individual patient samples (**Supplementary Figure 6**). Efficacy for (+)TDNT removal of positively charged cytokines (**Supplementary Figure 7A**), (−)TDNT removal of negatively charged cytokines (**Supplementary Figure 7B**), and (pan)TDNT and MG250^®^ removal of total cytokines (**Supplementary Figures 7C, D**) remained consistent irrespective of total cytokine burden. While the (+)TDNT and (−)TDNT displayed strong potential promise for selective cytokine removal based on charge, their overall efficacy for cytokine clearance was low (**Supplementary Figures 5E, F**). The smaller C4 hydrophobic groups in (+)TDNT and (−)TDNT allow more selective cytokine removal due to decreased nonspecific hydrophobic interactions; however, this comes at the cost of overall efficacy. Although we have demonstrated that cytokines can be selectively targeted based on their charge disparity, further optimization of the TDNT structure is needed to achieve an optimal balance between charge and hydrophobic interactions, improving overall cytokine clearance while maintaining charge-selective properties.

**Figure 6:**
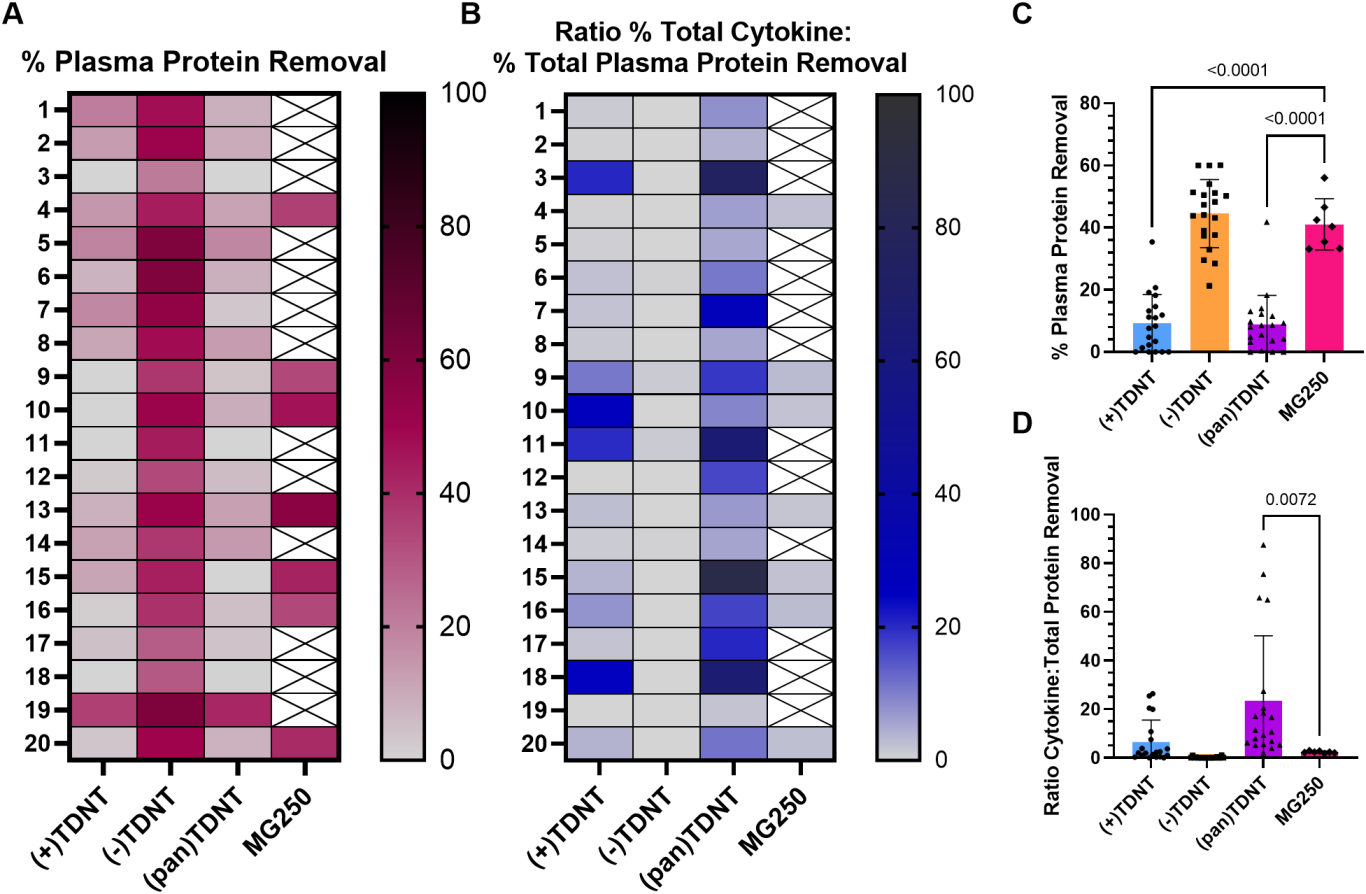
TDNT resins preferentially remove cytokines compared to plasma proteins from sepsis patient plasma. Heat map of **(A)** percentage of total plasma protein removed or **(B)** ratio of percentage of total cytokines removed to percentage of total plasma protein removed by TDNT resins compared to MG250 commercial control after 4 h of incubation with sepsis patient plasma. **(A, B)** White box with X indicates sample not tested. **(C)** Summary of the percentage of total plasma protein removed in **(A)**. **(D)** Summary of the ratio of the percentage of total cytokines removed to the percentage of total plasma protein removed in **(B)**. **(C, D)** Each symbol corresponds to an individual patient sample. Mean with error bars for SD represented on the graphs. Statistical significance was determined by one-way ANOVA with Dunnett’s multiple comparisons test. Significant *p*-values of < 0.05 are depicted on the graphs.

To evaluate the potential off-target effect of plasma incubation with TDNT, total plasma protein removal was assessed for comparison with cytokine removal. Both (pan)TDNT and (+)TDNT demonstrated significantly reduced removal of total protein from plasma compared to the commercial MG250^®^ control, whereas (−)TDNT demonstrated high plasma protein removal like MG250^®^ (**Figures 6A, C**). Removal of abundant plasma proteins during hemoperfusion is a significant safety concern because it can worsen tissue edema (17–21). The minimal serum protein adsorption by (pan)TDNT and (+)TDNT compared to MG250 indicates better biocompatibility. To further compare the selectivity of resins for cytokine removal relative to total plasma protein removal, the ratio of cytokine removal to total protein removal was evaluated. Compared to the commercial MG250^®^ control resin, (pan)TDNT exhibited a significantly higher ratio of cytokine to total protein removal from sepsis patient plasma (**Figures 6B, D**). This indicates that (pan)TDNT is a promising resin formulation, as it has greater selectivity for cytokine removal compared to conventional MG250^®^ hemoperfusion therapies. Furthermore, the adsorption of model antibiotics on TDNT resins was evaluated. For example, doxycycline adsorption occurred much faster in MG250 resin, with the saturated adsorption capacity approximately 12 times higher than that in (pan)TDNT (48 µg/mg vs. 4 µg/mg) (**Supplementary Figure 8**).

**Figure 7:**
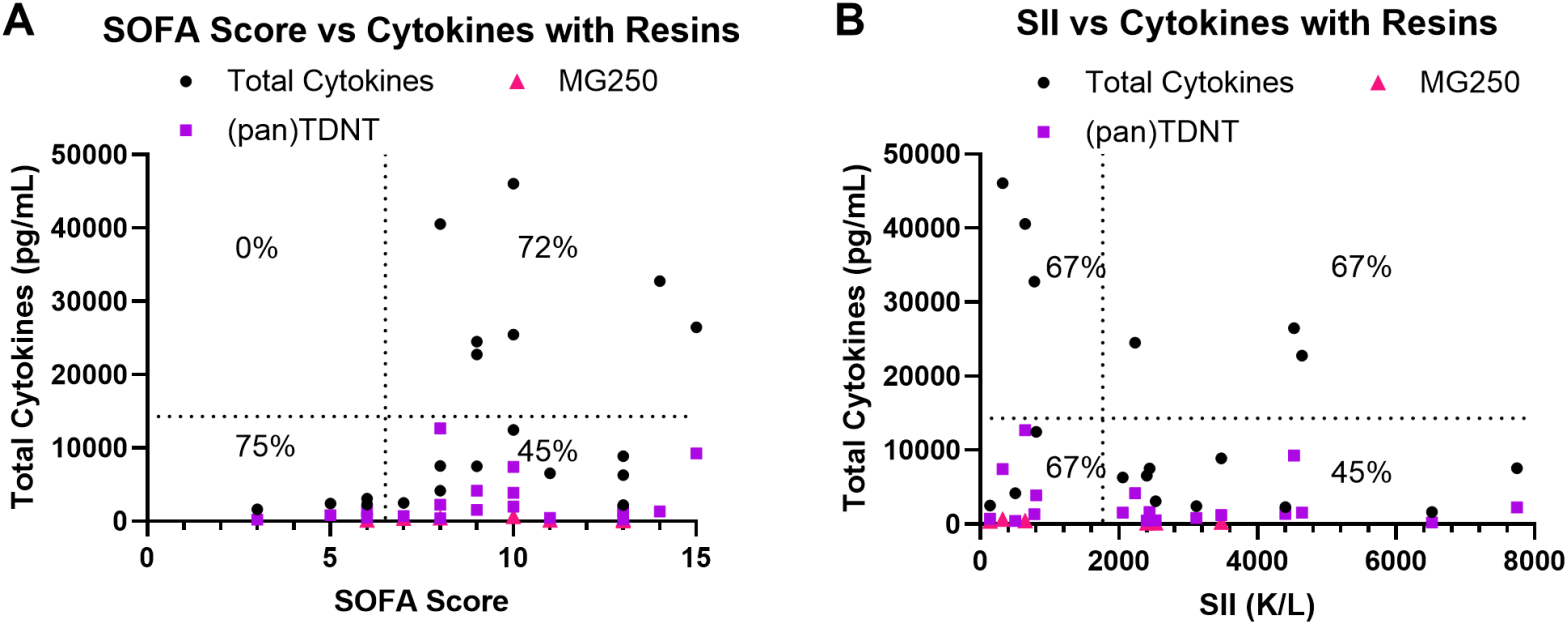
(pan)TDNT cytokine clearance efficiency by sepsis patient SOFA score and SII. **(A, B)** Total plasma cytokine levels (black) compared to cytokine levels after incubation with (pan)TDNT (purple) or commercial MG250^®^ (pink), showing the relationship with patient **(A)** SOFA score or **(B)** SII. Survival rates for patients in each domain are displayed in each quadrant of the graphs.

Due to the heterogeneity among sepsis patients, we examined whether cytokine removal might reduce the mortality rate in specific populations. When comparing cytokine levels in sepsis patient plasma before (black) and after incubation with (pan)TDNT (purple) or MG250^®^ commercial control (pink), similar efficacy was observed across all SOFA scores (**Figure 7A**) and all SII scores (**Figure 7B**) in the cohort. These results highlight the potential clinical benefit of reducing mortality in high-risk patient groups with high cytokine and high SOFA (72% mortality) or high SII (67% mortality) by removing excessive cytokines, thereby shifting these patients into the lower-risk, low-cytokine, and high-SOFA/SII population, which has a mortality rate of 45%. (pan)TDNT displayed comparable cytokine clearance efficacy regardless of total cytokine levels or the charge of cytokines present in the patient sample prior to resin incubation, as well as patient SOFA score and SII (**Supplementary Figure 9**). Although (pan)TDNT was effective across the range of SOFA and SII scores in the patient population, this finding highlights the need for more robust and reliable methods to define sepsis patient phenotypes for effective implementation of precise immune modulation therapies in the future. Many studies over the past decade have attempted to establish phenotypes and/or immunotypes for sepsis patients (52–55); however, a method for categorization that can be used reliably for clinical decision-making has yet to be developed. As efforts continue to optimize the promising TDNT resin platform for effective and selective removal of cytokines for precise immune modulation in sepsis, it is also critical to define a reliable method to determine which patient types will derive clinical benefit from this therapy.

## 4 Discussion

Despite current therapeutic interventions, sepsis mortality remains high (12), highlighting the urgent need for innovative treatment strategies to improve outcomes for these critically ill patients. Targeting cytokines to combat hyperinflammation in sepsis has been a sought-after strategy for decades, yet it has not provided significant clinical benefit. Therapeutics targeting individual cytokines, such as monoclonal antibodies (13, 14), offer a precise immune modulation approach; however, targeting a single inflammatory mediator is insufficient to address the complexity of immune dysregulation in sepsis to achieve clinical benefit. Conventional hemoperfusion approaches can attenuate certain inflammatory mediators but lack the flexibility to fine-tune adsorption profiles, as they rely on nonspecific hydrophobic interactions for adsorption. This lack of specificity may lead to clinical complications for sepsis management due to the nonspecific adsorption of antibiotics (61–64) and serum proteins in blood (17–21). In addition, inflammatory molecules, e.g., LPS and cytokines, adsorbed and immobilized on the surface of absorbents in hemoperfusion cartridges may continue to stimulate immune cells in circulation, thereby prolonging immune activation. This mechanism has been proposed to contribute to increased mortality in hemoperfusion therapy in certain apparently vulnerable subgroups (65). In contrast, our TDNT is immobilized in hydrogel polyethylene glycol resins, which have an optimal pore size (< 40 kDa) that allows medium-sized cytokines to diffuse in and be captured by TDNT within the hydrogel network (29). PEG is highly hydrophilic, reducing nonspecific protein/cytokine/toxin adsorption and preventing cell interaction. In addition, we can block the surface functional group on PEG resin before TD conjugation, as reported in our previous study (66), which prevents surface exposure of TD, eliminates the surface immobilization of immune-stimulating molecules, and reduces constant immune stimulation. Thus, the goal of this study was to develop the TDNT platform to provide effective and selective removal of cytokines, enabling a more precise immune modulation approach to improve outcomes in sepsis and address this gap in critical care. Recent advances have been made toward developing “point-of-care” tests for early sepsis detection that more rapidly and accurately profile patient immune status, thereby directing TDNT resin application to target the overflowing immunopathogenic cytokine profiles (67, 68).

Previously, we have demonstrated that therapeutics employing the TDNT platform show promising preclinical efficacy in attenuating hyperinflammation and improving survival in murine sepsis models (29, 30). This innovative strategy uses a combination of charged and hydrophobic moieties to selectively target cytokines based on the recently reported phenomenon of cytokine charge disparity, in which proinflammatory cytokines tend to have a negative charge, and anti-inflammatory cytokines tend to have a positive charge (29, 33). We recently reviewed the theoretical and experimental charges, post-translational modifications (PTMs), pharmacokinetics, biodistribution, and immune functions of cytokines and chemokines (33). Some cytokines can exert dual functions, displaying either pro- or anti-inflammatory properties depending on cell type and the spatial and temporal microenvironment. Therefore, it may be oversimplified to classify cytokines strictly into pro- or anti-inflammatory categories and to correlate their functions solely based on charge disparity. Exceptions are noted in the Yin-Yang chart in the review (33): a small group of anti-inflammatory cytokines, such as competitive IL-1 antagonists including IL-1RA, share similar sequences and negative charges with IL-1; conversely, a subset of proinflammatory cytokines can be positively charged, which may favor tissue and extracellular matrix (ECM) adhesion for localized immune stimulation (see the small circled groups in the Yin-Yang chart. Overall, major cytokines in innate immune responses generally follow the charge disparity trend; for example, negatively charged proinflammatory TNF-α, IL-1β, and IL-6, and positively charged anti-inflammatory IL-10, IL-4, and transforming growth factor beta (TGF-β). PTMs may modestly shift a cytokine’s net charge and are generally expected to maintain or reduce the *p*I, mainly via glycosylation and phosphorylation, respectively. In addition, some procytokines and membrane-bound cytokines are activated by enzymatic cleavage, which can alter (and in some cases reverse) the overall charge of the active cytokine. For example, proinflammatory IL-33 and IL-36β become more negatively charged after cleavage during activation. Importantly, experimentally measured *p*Is for most proinflammatory cytokines generally maintain a negatively charged profile (and vice versa for anti-inflammatory cytokines), indicating that the overall charge disparity trend is largely preserved despite PTMs. Taken together, cytokine charge disparity provides a practical design principle for selective capture, but it should be interpreted as a dominant trend rather than an absolute classification rule.

To promote successful clinical translation, we have generated a library of TDNT formulations with various charged and hydrophobic groups at different densities and valencies (**Supplementary Figure 1**) to identify and validate the most promising candidates for effective and selective removal of cytokines in sepsis patient plasma (**Figure 1**). Initial evaluation of model protein capture using the TDNT resin library revealed that TDNT formulations with positively charged moieties or a combination of positively charged and hydrophobic moieties preferentially loaded negatively charged αLA, whereas TDNT formulations with negatively charged moieties or a combination of negatively charged and hydrophobic moieties preferentially loaded positively charged lysozyme (**Supplementary Figures 2**, **3**). TDNT formulations with only hydrophobic moieties did not display charge-selective protein capture and had overall low efficacy for model protein loading, indicating the benefit of incorporating charged groups into the TDNT structure for improved protein loading. These results confirm the validity of the TDNT approach for effective and selective capture of proteins based on charge and justify further evaluation using biological samples.

Cytokine levels in sepsis patients’ blood are generally very heterogeneous, with some cytokines present at low picogram-per-milliliter levels that cannot be reliably measured before or after adsorption. Thus, we doped mixed patient plasma with inflamed THP-1 cell culture medium to ensure the presence of a broader spectrum of cytokines with abundant levels for screening multiple TDNT resins. However, during verification of the lead TDNTs with individual patient plasma, no cell culture medium was added to ensure the cytokine profile reflected individual patient status. Spiked-mixed sepsis patient plasma was used for library screening to ensure the presence of an abundance of cytokines to effectively evaluate TDNT cytokine removal efficacy, as there is significant cytokine profile heterogeneity among individual sepsis patients, even with a similar septic source in the cohort (**Figure 3**). We identified 100% PEGA-OA_4_C17_4_ as the lead (pan)TDNT, 100% PEGA-Arg_4_C4_4_ as the lead (−)TDNT, and 100% PEGA-COOH_4_C4_4_ as the lead (+)TDNT (**Figure 1**). Further evaluation of these lead candidates in individual sepsis patients confirmed the selective binding profiles of (−)TDNT and (+)TDNT and the high efficacy of (pan)TDNT when compared with commercial MG250^®^ resin (**Figures 4**–**6**; **Supplementary Figures 4**–**6**), irrespective of total plasma cytokine burden (**Supplementary Figure 7**). While (−)TDNT and (+)TDNT displayed charge-selective binding profiles among the cohort of sepsis patient plasma samples, their overall efficacy must be further improved through engineering optimization. The short C4 hydrophobic groups allow stronger charge interactions to promote increased charge selectivity compared with larger hydrophobic groups (e.g., C17); however, this occurs at the expense of efficacy. Thus, the balance between charge and hydrophobic interactions must be optimized to provide an optimal charge-selective and effective cytokine-binding profile.

The superior selectivity of (pan)TDNT for cytokine removal compared with other plasma proteins (**Figure 6**), relative to commercial MG250^®^ resin, indicates that this TDNT formulation has more promising clinical potential for sepsis treatment than traditional hemoperfusion therapies because of the decreased potential for off-target side effects, such as edema from the loss of plasma protein (17–21). However, the potential nonspecific adsorption of small signaling proteins, e.g., insulin and other hormones, in TDNT resin needs to be further characterized. Negatively charged cytokines may more often exist as free proteins in biofluids, given that the most abundant carrier proteins are overall negatively charged and may impose charge repulsion. In contrast, positively charged anti-inflammatory cytokines (e.g., IL-10, TGF-β) and many chemokines are known to associate with negatively charged carrier proteins (e.g., albumin), heparin, and the extracellular matrix, which favor localized inflammation control and immune cell chemotaxis, respectively. Although carrier binding may still alter the effective kinetics of eliminating positively charged cytokines from the blood circulation during hemoperfusion, these interactions are not expected to change the intrinsic charge selectivity of our approach. The size-exclusion effect of the hydrogel resin primarily allows small, free cytokines to diffuse into the network and be captured by the TD nanotrap. Therefore, we designed our experiments to evaluate cytokine adsorption in whole plasma to better support translation of these findings to *in vivo* efficacy and clinical applications.

Conventional hemoperfusion approaches can also reduce plasma levels of antibiotics by hydrophobic interaction (61–64), which can be reduced by the hydrophilic TDNT PEGA resin, as shown in doxycycline adsorption (**Supplementary Figure 8**). However, some charged antibiotics (e.g., polymyxin B, daptomycin), vitamins, and small signaling proteins, e.g., insulin, also present charge and hydrophobic features that allow interaction with TDNT. Although proteins/cytokines outcompete small molecules/peptides for TDNT binding because of multivalent effects, these critical biomolecules and drugs used in sepsis treatment need to be further monitored both *in vitro* and *ex vivo* during TDNT hemoperfusion treatment in comparison with commercially/clinically used hemoperfusion resins.

There were some technical limitations in the current study. While the TD nanotrap platform demonstrates great potential in patient plasma incubation, the *in vitro* screening methodology has several limitations. Although the TDNT hydrogel resins demonstrate high biocompatibility in cell culture (noncytotoxic and noncell adhesion) and in whole blood incubation (nonhemolytic and nonimmunogenic), further evaluation of TDNT in whole blood incubation is needed to assess the potential impact of resin–cell and cell–cytokine interactions on cytokine adsorption efficiency. Furthermore, the dynamic flow of whole blood through a cartridge packed with TDNT resin could better mimic the temporal and spatial cytokine diffusion and adsorption observed in extracorporeal hemoperfusion treatments. The hemoperfusion approach can only gradually absorb overflowing cytokines in the blood circulation and cannot directly eliminate local high concentrations of cytokines within tissues and organs to control the source of inflammation. Rather, it prevents the spread of hyperinflammation and protects against remote organ damage. We may combine TDNT hydrogel resin hemoperfusion therapy with the injectable TDNT nanogel (200–300 nm) (30) to target both overflowing deep-tissue and circulating cytokines for a more effective immune modulation in sepsis treatment.

Huge efforts have been invested in developing immune-modulating approaches, and hundreds of clinical trials have been conducted for sepsis treatments; however, none have successfully reduced sepsis mortality. The paradigm of hyperinflammation causing sepsis has been challenged (69) with the argument regarding the “cause-and-effect” relationship of hyperinflammation in sepsis, i.e., cytokine storm. Cytokine levels in sepsis patients are dynamic and highly heterogeneous. Continuous cytokine monitoring in sepsis patients is challenging. The early cytokines, e.g., TNF-α, may already have passed their peaks after the diagnosis of sepsis in the clinic. Excessive cytokine production and dysregulation contribute to sepsis development and organ damage. (pan)TDNT demonstrated high cytokine clearance efficacy irrespective of cytokine levels, SOFA score, and SII status (**Figure 7**), which may be beneficial for treating the early state of sepsis or patients in the hospital at high risk of developing sepsis or cytokine release syndrome, e.g., severe cardiac surgery, pancreatitis, and CAR-T therapy. However, it is not clear whether specific cytokines or combinations of cytokines, as prognostic biomarkers, continue to play fatal roles in the later stage of sepsis and septic shock. The lack of readily available and reliable biomarkers to predict therapeutic efficacy remains a significant challenge for future clinical investigation and implementation, as it is for many other therapeutic interventions (70, 71). Prompt and accurate diagnosis of patient immune status and cytokine profiles is needed to determine the use of TDNT therapy to target selective cytokine profiles. In addition, the sepsis patient population in this study was limited to surgical abdominal sepsis, and the sample size was relatively small. TDNT nanogel needs to be further optimized for both selective and effective cytokine adsorption in blood samples from larger cohorts and a more heterogeneous population of sepsis patients.

In conclusion, we have demonstrated that TDNT can be engineered to remove cytokines effectively and selectively from sepsis patient plasma based on the cytokine charge disparity, as primarily shown with (−)TDNT and (+)TDNT resins. The TD structure can be further optimized to balance the charge and hydrophobic moieties for improved efficacy. Additionally, the (pan)TDNT demonstrated comparable efficacy and higher specificity for cytokine removal compared to classical commercial macroporous adsorption resin, with reduced nonspecific adsorption of antibiotics and serum proteins. In addition, MG250^®^ exhibited unbalanced cytokine adsorption, e.g., complete IL-1RA adsorption alongside moderate/low adsorption of IL-1α, IL-1β, and IL-18, which could exacerbate inflammation in some patients, whereas (pan)TDNT provided more balanced immune modulation in terms of IL-1 family cytokine removal. Finally, the pan-affinitive TDNT resin consistently reduced a broad panel of cytokines in septic patient plasma *ex vivo* across the range of SOFA and SII scores evaluated in this cohort, resulting in lower overall cytokine burden. These findings are preliminary and hypothesis-generating, and their clinical significance will require validation in larger, more diverse sepsis cohorts. Charge-based cytokine profiling and total cytokine burden in patient blood may represent a novel theragnostic marker for sepsis treatment.

## Data Availability

The original contributions presented in the study are included in the article/**Supplementary Material**. Further inquiries can be directed to the corresponding author.
