# Supplementary material for: Telodendrimer nanotrap for selective cytokine removal from sepsis patient plasma
Source: Front Immunol. 2026 Mar 25;17:1633723. doi: 10.3389/fimmu.2026.1633723 (PMC13056833; doi:10.3389/fimmu.2026.1633723)
Supplement: Supplementary file 1 [file DataSheet1.docx]

**Supplemental Information Telodendrimer nanotrap for selective cytokine removal from sepsis patient plasma.**

**Jennifer M. Messina^1ϯ^, Dandan Guo^1ϯ^, Changying Shi^1^, Natalia Valenzuela^1^, Qinghe Meng^2,3^, Robert N Cooney^2,3*^, and Juntao Luo^1,2,3,4,5**^**

^1^ Department of Pharmacology, State University of New York Upstate Medical University, Syracuse, NY, United States

^2^ Department of Surgery, State University of New York Upstate Medical University, Syracuse, NY, United States

^3^ Upstate Sepsis Interdisciplinary Research Center, State University of New York Upstate Medical University, Syracuse, NY, United States

^4^ Department of Microbiology and Immunology, State University of New York Upstate Medical University, Syracuse, NY, United States

^5^ Upstate Cancer Center, State University of New York Upstate Medical University, Syracuse, NY, United States

**^ϯ^**These authors contributed equally to this work and share first authorship.

***Correspondence:** [luoj@upstate.edu](mailto:luoj@upstate.edu), CooneyR@upstate.edu

**Chemical Reagents**

Poly[acryloyl-bis(aminopropyl)polyethylene glycol] (PEGA) resins (50-100 mesh, 0.45 mmol/g) were purchased from Nanjing RAWPEG biotechnology Limited. (Fmoc)Lys(Fmoc)-OH, (Fmoc)Lys(Boc)-OH, and (Fmoc)Arg(Pbf)-OH were purchased from Chem-Impex International Inc. (Wood Dale, IL). 4-carboxyphenylboronic acid was obtained from Combi-Blocks Ins (San Diego, CA). N,N’-diisopropylcarbodiimide (DIC), N-hydroxybenzotriazole (HOBt), 4-methylpiperidine, dimethyl-sulfoxide (DMSO), dimethylformamide (DMF), methyl ether, dichloromethane (DCM), methanol, methyl oxalyl chloride, succinic anhydride, 4-methypiperidin, lithium hydroxide (LiOH), acetic anhydride (OAc), butyric acid (C4) , Propylpentanoic acid (PPA), 4-phenylbutyric acid (PBA), nonanoic acid (C9), heptadecanoic acid (C17) and all other chemicals were purchased from Sigma-Aldrich and Acros organics and used without further purification. Cyanine3-NHS ester (Cy3-NHS) was purchased from AAT Bioquest, Pleasanton, CA. alpha-lactalbumin (α-LA,Bovine milk, was purchased from USBiological. Lysozyme was purchased from MP biomedicals, Solon, OH.

**Telodendrimer Nanotrap Resin Engineering and Synthesis**

Following our previous procedure(1), we apply PEGA resin to synthesize TD NT with diverse charge and hydrophobic combinations (**Fig 1A and S1**) via Fmoc peptide chemistry. Before TD synthesis, the functional group loading capacity in a batch of NH_2_-PEGA resin was down scaled by acetic anhydride after partially 10% temporary blocking with (Fmoc)-Gly-OH following our previous publications. (2, 3) After De-Fmoc, these 10% NH_2_-PEGA resin will be applied for TDNT synthesis in parallel with 100% NH_2_-PEGA resin. (Fmoc)-Lys(Fmoc)-OH and Fmoc-Oligo (ethylene glycol)-COOH linker were coupled sequentially following the standard peptide synthesis procedures. DIC and HOBt were used as catalytic coupling reagents. All reactants were in 3-fold excess with respect to the amine functional group on resin. After second generation dendritic oligolysine synthesis, (Fmoc)-Arg(Pbf)-OH or (Fmoc)-Lys(Boc)-OH were used to introduce the third layer of oligolysine on PEGA resin to introduce the orthogonally protected amine groups for charge and hydrophobic moiety conjugation. De-Fmoc was carried out in 20% 4-methylpiperidine DMF solution for 30 min. Pbf protecting group was removed in the presence of TFA/DCM (50/50, v/v) for 2 h. After the completion of each step reaction, residual reactants were removed under vacuum and washed with copious solvents of DMF, DCM, and MeOH sequentially. The protein-binding hydrophobic building blocks were conjugated on the α-amine on the arginine or lysine after de-Fmoc step via the standard peptide synthesis procedure. At the end, TD NT resins were washed with organic solvents and water, PBS gradually for readily model protein and cytokine adsorptions.

**In vitro model protein removal assay**

To screen TD NT formulations for protein removal efficacy, two abundant low molecular weight proteins, one with negative charge (isoelectric point (pI)<7.4) and one with positive charge (pI>7.4), were selected to mimic different charged cytokines. Alpha-lactalbumin, (αLA), from bovine milk, (MW 14.2 kDa, pI 4.8) (Sigma-Aldrich) served as a negatively charged model protein and lysozyme (MW 14 kDa, pI 11.4) (MP Biomedicals) served as a positively charged model protein. Both αLA and lysozyme were conjugated to fluorescent dye cyanine 3 (CY3) as previously reported(4). Briefly, for preparation of Cy3- αLA, Cyanine3-NHS ester (Cy3-NHS) (AAT Bioquest, Pleasanton, CA) dissolved 200 µL dimethyl sulfoxide (DMSO) was added into a flask with α-LA (Bovine milk, USBiological) dissolved 0.1 M NaHCO_3_ (pH 8.5) solution at 1:1 molar ratio and covered with aluminum foil for light-protection. After 24 h reaction at 4°C in the cold room, the reaction solution was transferred to a dialysis tubing (Spectra/Pro®, molecular weight cut-off (MWCO) 3.5k) and further purified by dialysis against deionized water for 3 days in the dark at 4°C with frequently refresh the deionized water to remove the unreacted Cy3 NHS ester. The Cy3-αLA powder was obtained using lyophilization. Gel electrophoresis was used to confirm the negatively charged property of Cy3-αLA via 1.5% agarose gel under 40 v at 40 min (data not shown). For preparation of Cy3-lysosyme, Cyanine3-NHS ester (Cy3-NHS) (AAT Bioquest, Pleasanton, CA) dissolved 200 µL dimethyl sulfoxide (DMSO) was added into a flask with lysozyme (MP biomedicals, Solon, OH) dissolved 0.1 M NaHCO_3_ (pH 8.5) solution at 1:1 molar ratio and covered with aluminum foil for light-protection. After 24 h reaction at 4°C in the cold room, the reaction solution was transferred to a dialysis tubing (Spectra/Pro®, molecular weight cut-off (MWCO) 3.5k) and further purified by dialysis against deionized water for 3 days in the dark at 4°C with frequently refresh the deionized water to remove the unreacted Cy3 NHS ester. The Cy3-Lyso powder was obtained using lyophilization. Gel electrophoresis was used to confirm the positively charged property of Cy3-Lyso via 1.5% agarose gel under 40 v at 40 min,as shown in our previous studies. (5).

TD NT resins were stored in sterilized PBS solution at 4 °C in the refrigerator. After PBS solution was drained by vacuum, 20±2 mg wet resins were weighted in Eppendorf tubes. 90 µL PBS solution was then added to the Eppendorf tubes with the resins. 10 µL of 10 mg/mL Cy3-αLA or Cy3-lysozyme were added into each resin-containing Eppendorf tubes, vortex to mix resin and solution, spined down the resins by centrifugation (1000 rpm, 3 min) and incubated at room temperature on the tube rotator (Fisher Scientific) for ~18 h overnight. The control solution of Cy3-αLA or Cy3-lysozyme was made of 10 µL of 10 mg/mL Cy3-α-LA or Cy3-Lyso added into 90 µL PBS solution without resins. The fluorescence intensity of Cy3-αLA or Cy3-Lyso in the supernatant after incubation with the resins were used to evaluate the protein capture capacity of the resins. The fluorescence intensity of supernatant and control solution were determined by microplate reader (BioTek Synergy H1) with excitation of 550 nm and emission of 580 nm. Each test was performed in duplicate. Percent capture efficiency was calculated using the following equation:

$\% Capture Efficency= \frac{{RFU}_{original}-{RFU}_{remaining}}{{RFU}_{original}}x100$.

Ratios of % capture efficiency of αLA:lysozyme and lysozyme:αLA were calculated for each resin (**Fig S3**) to compare selectivity for negative charge and positive charge protein capture, respectively.

**Results**

**Engineering of Telodendrimer Nanotrap Resins for Protein Removal**

The combination of charge and hydrophobic interactions is critical for effective protein delivery and capture by TD nanotrap. Although the charge interactions can be selective, the hydrophobic interactions are nonspecific for protein interaction. Thus, the balance between charge and hydrophobic interactions is essential to yield both effective and selective cytokine removal based on charge disparity. Additionally, the introduction of hydrophobic moieties into hydrophilic PEGA resin during TD synthesis may induce shrinkage of resin in aqueous solution depending on the TD density. Thus, we have engineered TD density on PEGA resin to fine tune the capture efficiency of protein, cytokines, and drug molecules in TD nanotrap(2, 3). In this study, we selected several hydrophobic moieties as shown in **Fig 1A**, three charged groups and two different TD density to optimize the selectivity of nanotrap for cytokine removal. Following our previous procedure, TD was synthesized on PEGA resin via solid phase peptide synthesis via HOBT/DIC chemistry(2, 3). In addition to arginine and carboxylic acid, we chose oxalic acid (OA) for comparison. Given the strong affinity to bivalent cations, e.g. calcium, OA may serve as a dual affinitive motif for both positively and negatively charged cytokine interactions, which also increases the hydrophilicity of TD to sustain the swelling property and pore sizes of PEGA resin for effective cytokine diffusion and capture. According to our previous studies, high TD density in PEGA, especially for hydrophobic-rich TD constructs, causes the hydrogel resin shrink in water. This shrinkage can be avoided by reducing the TD density, as demonstrated by improved efficacy for model protein adsorption by PEGA resin with some hydrophobic TD at 1% of original resin capacity (reduced to 0.004 mmol/g)(2, 3). Accordingly in this study, we also down-scaled the functional group capacity in PEGA resin to 10% (0.04 mmol/g) of original capacity via partial amine blockage before TD synthesis to compare the efficacy and selectivity for protein and cytokine removal with the high density PEGA-TD resins (0.4 mmol/g).

**TD NT Resins Display Charge Selective Capture of Model Proteins**

TD NT resin adsorption of protein is dependent on the combination of protein properties, such as protein size, charge, and hydrophobicity, and properties of the TD NT in the PEGA resin, such as chemical structure, valency, and density(2, 3). Thus, our library of TD NT resins includes those with charged only, hydrophobic only, and charged and hydrophobic moieties at various densities and valences (**Fig S1**) to optimize the capture of small, charged proteins for effective sequestering of cytokines. Recently, we have reported on the phenomenon of cytokine charge disparity in which the majority of pro-inflammatory cytokines have a negative charge, while the majority of anti-inflammatory cytokines have a positive charge(1, 6), thus inspiring our efforts to target charge differences amongst cytokines for a more precise immune modulation in sepsis. During TD NT screening for protein adsorption, two abundant low molecular weight proteins with isoelectric point (pI) either higher or lower than neutral pH 7.4 to mimic cytokines with the medium sizes and different charges: Alpha-lactalbumin (αLA) from bovine milk, (MW 14.2 kDa, pI 4.8, Sigma-Aldrich) was selected as a negatively charged model protein to mimic key proinflammatory cytokines (TNFα, IL-1β, IL-6, etc.) ; and lysozyme (MW 14 kDa, pI 11.4, MP Biomedicals) served as a positively charged model protein to mimic anti-inflammatory cytokines (IL-10, IL-4, etc). αLA and lysozyme were conjugated to fluorescent dye Cy3 so that protein capture into TD NT resins could be monitored by fluorescence spectrometry (**Fig 1B**). PEGA without TD NT conjugation served as a negative control for protein capture.

The positively charged TD NT resins with or without hydrophobic moieties both exhibited better efficiency in adsorbing negatively charged αLA compared to positively charged lysozyme (**Fig S2 A+D**). Accordingly, the positively charged TD NT resins with or without hydrophobic moieties generally displayed a loading ratio of αLA:lysozyme greater than 1 (**Fig S3A**), and conversely a capture ratio of lysozyme:αLA of less than 1 (**Fig S3B**), indicating charge selective capture of negatively charged protein by these resins. In TD NT resins with only positively charged moieties, resins with 100% density more efficiently loaded negatively charged αLA (**Fig S2 A**) and displayed greater charged selectivity for protein capture (**Fig S3**) compared to those with 10% density. TD NT resins with both positively charged and hydrophobic moieties showed a similar trend (**Fig S2 D** and **Fig S3**), with a few exceptions, e.g. PEGA-Arg_2_C17_2_ and PEGA- Arg_4_C17_4._ The bulky hydrophobic 17 carbon fatty acid tails resulted in decreased protein adsorption and charge selectivity of TD NT resins in comparison to the smaller hydrophobic moieties such as PEGA- Arg_4_C4_4_ and PEGA- Arg_4_C9_4_. Additionally, PEGA-Arg_2_C17_2_ and PEGA- Arg_4_C17_4_ load protein more efficiently at 10% density compared to 100% density, unlike TD NT resin formulations with the smaller hydrophobic moieties. This is due to both reduced resin swelling, which prevents protein diffusion, and increased nonspecific hydrophobic interactions, which reduces charge selectivity for protein capture in these TD NT resins with both bulky hydrophobic groups at high density.

As expected, the negatively charged TD NT resins with or without hydrophobic moieties adsorbed positively charged lysosome more effectively compared to negatively charged αLA (**Fig S2 B+E**), with a general capture ratio of αLA/lysozyme less than one (**Fig S3A**), and conversely a capture ratio of lysozyme:αLA of greater than 1 (**Fig S3B**), indicating charge selective capture of positively charged protein by these resins. Like the TD NT resins with positively charged moieties, TD NT resins with only negatively charged moieties load protein not only more efficiently but also with more charge selectivity in 100% density resin than those with 10% density (**Fig S2 B**). Surprisingly, most of TD NT resins with both negative charges and hydrophobic moieties showed even greater charge selectivity for capture of positively charged protein compared to TD resins with only negative charges (**Fig S3**). Interestingly, a few oxalic acid containing TD NT resins with long fatty acid tails, ex. PEGA-OA_2_C17_2_, PEGA-OA_4_C17_4,_ and PEGA-COOH_4_C17_4_, showed significant capture of negatively charged αLA (**Fig S2 E** and **Fig S3**), which may serve as pan-affinitive TD NT resins to target all overflowing cytokines. As a general observation, resins with only hydrophobic groups did not show either effective protein capture nor charge selectivity towards αLA and lysozyme model proteins (**Fig S3**).

PEGA-OA_4_

PEGA-OA_2_

**A**

**B**

PEGA-OA_8_

PEGA-OA_4_OAc_4_

PEGA-COOH_4_OAc_4_

PEGA-Arg_4_OAc_4_

PEGA-Arg_2_NH2_2_

PEGA-Arg_4_NH2_4_

PEGA-Arg_8_NH2_8_

PEGA-C17_2_

PEGA-C17_4_

PEGA-C17_8_

PEGA-C4_4_

PEGA-C9_4_

PEGA-PPA_4_

PEGA-PBA_4_

**C**

**D**

PEGA-Arg_4_C17_4_

PEGA-Arg_4_C9_4_

PEGA-Arg_4_C4_4_

PEGA-Arg_4_PPA_4_

PEGA-Arg_4_PBA_4_

PEGA-Arg_2_C17_2_

PEGA-(NH_2_)_4_C17_4_

PEGA-Arg_4_VE_4_

PEGA-OA_4_C17_4_

PEGA-OA_2_C17_2_

**E**

PEGA-OA_4_C9_4_

PEGA-OA_4_C4_4_

PEGA-OA_4_PPA_4_

PEGA-OA_4_PBA_4_

PEGA-COOH_4_PPA_4_

PEGA-COOH_4_PBA_4_

PEGA-COOH_4_C9_4_

PEGA-COOH_4_C4_4_

PEGA-COOH_4_C17_4_

**Figure S1. Telodendrimer Nanotrap Resin Structures.** TD NT library consisting of PEGA based resins with **(A)** Positive Charged Only, **(B)** Negative Charged Only, **(C)** Hydrophobic Only, **(D)** Positive Charged and Hydrophobic, or **(E)** Negative Charged and Hydrophobic structural moieties.

**Figure S2. TD NT Resin *In Vitro* Model Protein Loading Efficiency** Measurement of protein loading of negatively charged αLA and positively charged lysozyme in TD NT resins with either 10% or 100% density and (**A**) only positively charged moieties, (**B**) only negatively charged moieties, (**C**) only hydrophobic moieties, (**D**) positive charged and hydrophobic moieties, or (**E**) negative charged and hydrophobic moieties. PEGA alone without TD NT serves as a negative control. Samples were run in duplicate. Mean with error bars for SD represented on the graphs.

**Figure S3. TD NT Resin Charge Selectivity for Model Proteins** Ratio of (**A**) αLA:Lysozyme and (**B**) Lysozyme:αLA percent capture efficiency. Ratio > 1 indicates a resin has a (**A**) negative protein-selective or (**B**) positive protein-selective adsorption profile. Ratio = 1 indicates no charge selectivity for protein adsorption. PEGA alone without TD NT serves as a negative control. Samples were run in duplicate.

**Figure S4. TD NT Positive Charged Cytokine Clearance Efficacy in Sepsis Patient Plasma.** Summary of (+)TDNT, (-)TDNT, (pan)TDNT and commercial MG250 removal of 6 positive charged cytokines (pI>7.4) from sepsis patient plasma: IL-7, IL-8, IL-10, IL-13, IL-17A, MCP-1. Each symbol corresponds to an individual patient sample. Mean with error bars for SD represented on the graphs. Statistical significance was determined by One-way ANOVA with Dunnett’s multiple comparisons test. Significant P-values of < 0.05 are depicted on the graphs.

**Figure S5. TD NT Negative Charged Cytokine Clearance Efficacy in Sepsis Patient Plasma.** Summary of (+)TDNT, (-)TDNT, (pan)TDNT and commercial MG250 removal of 9 negative charged cytokines (pI<7.4) from sepsis patient plasma: GMCSF, IL-1α, IL-1β, IL-1RA, IL-6, IL-18, MIP-1α, MIP-1β, TNFα. Each symbol corresponds to an individual patient sample. Mean with error bars for SD represented on the graphs. Statistical significance was determined by One-way ANOVA with Dunnett’s multiple comparisons test. Significant P-values of < 0.05 are depicted on the graphs.

**Figure S6. TD NT Resin Cytokine Charge Selectivity in Individual Patients. (A+B)** Heat maps to compare (+)TDNT, (-)TDNT, and (pan)TDNT resin clearance efficacy of total, negative charged, and positive charged cytokines in 20 individual sepsis patient plasma samples. Cytokine clearance efficacy is indicated by number in heap map boxes. Patient SOFA score and SII are indicated above the corresponding heat map. UA = SII unavailable. **(B)** Commercial MG250 resin (positive control) was evaluated for cytokine clearance efficacy in addition to TDNT resins in 7 plasma samples for comparison.

**Figure S7. TD NT Resin Cytokine Clearance Efficacy Relative to Total Plasma Cytokine Burden. (A)** (+)TDNT cytokine clearance efficacy of positive charged cytokines compared to total positive charged plasma cytokine burden [pg/mL]. **(B)** (-)TDNT cytokine clearance efficacy of negative charged cytokines compared to total negative charged plasma cytokine burden. **(C)** (pan)TDNT and **(D)** commercial MG250 total cytokine clearance efficacy compared to total plasma cytokine burden. Each symbol corresponds to an individual patient sample (**A-C** n=20 and **D** n=7).


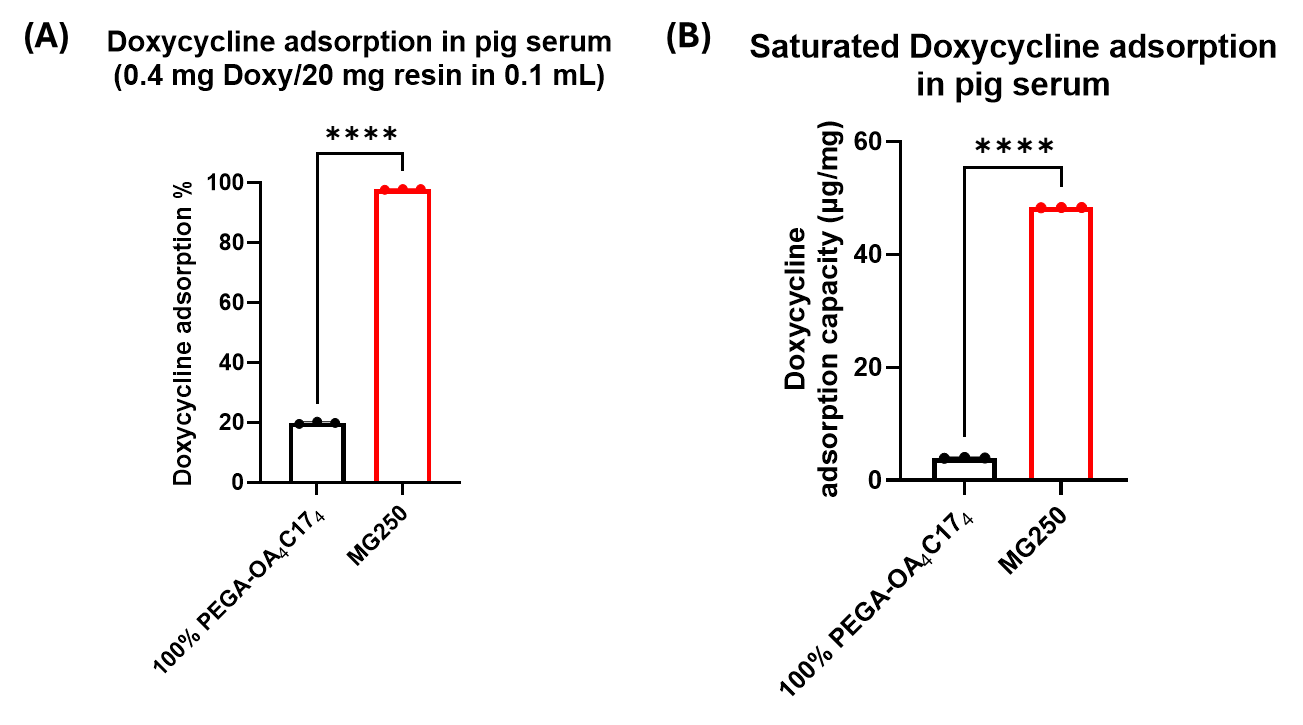
**Figure S8** Doxycycline adsorption in pan resin and MG250 in the pig serum. (A) Doxycycline adsorption in pan TDNT PEGA-OA_4_C17_4_ resin (20 mg) and MG250 resin (20 mg) after 1 h incubation 4 mg/mL of doxycycline in pig serum. (B) Saturated doxycycline adsorption capacity of pan TDNT PEGA-OA_4_C17_4_ resin and MG250 in pig serum.

**Figure S9. (pan)TDNT Cytokine Clearance Efficiency by Sepsis Patient Type.** Comparison of (pan)TDNT total cytokine clearance efficacy amongst patients with **(A)** low (<50^th^ percentile by weight) and high (>50^th^ percentile by weight) cytokine profiles, **(B)** low (<6.5) and high (>6.5) SOFA scores, and **(C)** low (<1767) and high (>1767) SII. Patients were also divided into categories based on a combination of low and high **(D)** SOFA score and cytokine, **(E)** SII and cytokine, and **(F)** SOFA score and SII for comparison of (pan)TDNT cytokine clearance efficacy.

**References**

1. Shi C, Wang X, Wang L, Meng Q, Guo D, Chen L, et al. A Nanotrap Improves Survival in Severe Sepsis by Attenuating Hyperinflammation. *Nature Communications* (2020) 11(1). doi: 10.1038/S41467-020-17153-0.

2. Yang X, Guo D, Ji X, Shi C, Luo J. Engineering Nanotrap Hydrogel for Immune Modulation in Wound Healing. *Macromol Rapid Commun* (2023) 44(23):e2300322. Epub 20230815. doi: 10.1002/marc.202300322.

3. Yang X, Guo D, Ji X, Shi C, Messina JM, Suo L, et al. Telodendrimer Functionalized Hydrogel Platform for Sustained Antibiotics Release in Infection Control. *Acta Biomaterialia* (2024) 178:147-59. doi: <https://doi.org/10.1016/j.actbio.2024.02.041>.

4. Wang X, Shi C, Zhang L, Bodman A, Guo D, Wang L, et al. Affinity-Controlled Protein Encapsulation into Sub-30 Nm Telodendrimer Nanocarriers by Multivalent and Synergistic Interactions. *Biomaterials* (2016) 101:258-. doi: 10.1016/J.BIOMATERIALS.2016.06.006.

5. Ji X, Yang X, Shi C, Guo D, Wang X, Messina JM, et al. Functionalized Core–Shell Nanogel Scavenger for Immune Modulation Therapy in Sepsis. *Advanced Therapeutics* (2022) 5(10):2200127. doi: <https://doi.org/10.1002/adtp.202200127>.

6. Messina JM, Luo M, Hossan MS, Gadelrab HA, Yang X, John A, et al. Unveiling Cytokine Charge Disparity as a Potential Mechanism for Immune Regulation. *Cytokine & Growth Factor Reviews* (2024) 77:1-14. doi: <https://doi.org/10.1016/j.cytogfr.2023.12.002>.
